# P2RX7 inhibitor suppresses exosome secretion and disease phenotype in P301S tau transgenic mice

**DOI:** 10.1186/s13024-020-00396-2

**Published:** 2020-08-18

**Authors:** Zhi Ruan, Jean-Christophe Delpech, Srinidhi Venkatesan Kalavai, Alicia A. Van Enoo, Jianqiao Hu, Seiko Ikezu, Tsuneya Ikezu

**Affiliations:** 1 Department of Pharmacology & Experimental Therapeutics, Boston University School of Medicine, 72 East Concord St, L-606B, Boston, MA 02118 USA; 2 Alzheimer’s Disease Center, Boston University School of Medicine, Boston, MA 02118 USA; 3 Center for Systems Neuroscience, Boston University, Boston, MA 02118 USA; 4 Neurology, Boston University School of Medicine, 72 East Concord St, L-606B, Boston, MA 02118 USA

**Keywords:** Alzheimer’s disease, Exosome, Extracellular vesicles, Microglia, Microtubule-associated protein tau, P2X purinoceptor 7, Tauopathy

## Abstract

**Background:**

Neuronal accumulation of misfolded microtubule-associated protein tau is a hallmark of neuropathology in Alzheimer’s disease, frontotemporal dementia, and other tauopathies, and has been a therapeutic target. Microglia can spread tau pathology by secreting tau-containing exosomes, although the specific molecular target is yet to be identified for the therapeutic intervention. P2X purinoceptor 7 (P2RX7) is an ATP-gated cation channel, enriched in microglia and triggers exosome secretion. The purpose of the study is to examine the therapeutic effect of an orally applicable, CNS-penetrant P2RX7 specific inhibitor on the early disease stage of a tauopathy mouse model.

**Methods:**

Three-months-old P301S tau mice were treated with P2RX7-specific inhibitor GSK1482160 or vehicle for 30 days, followed by behavioral, biochemical and immunohistochemical assessment. GSK1482160 was also tested for exosome secretion from primary cultured murine astrocytes, neurons and microglia in vitro.

**Results:**

Oral administration of GSK1482160 significantly reduced accumulation of MC1^+^ and Alz50^+^ misfolded tau in hippocampal regions, which was accompanied with reduced accumulation of Tsg101, an exosome marker, in hippocampal neurons. Proximity ligation assay demonstrated complex formation of Alz50^+^ tau and Tsg101 in hippocampal neurons, which was reduced by GSK1482160. On the other hand, GSK1482160 had no effect on microglial ramification or CD68 expression, which was significantly enhanced in P301S mice, or pro/anti-inflammatory cytokine gene expression. Strikingly, GSK1482160-treated P301S mice show significantly improved working and contextual memory as determined by Y-maze and fear conditioning tests. GSK1482160 also significantly increased accumulation of Tsg101 and CD81 in microglia in vivo*,* suggesting its suppression of P2RX7-induced exosome secretion from microglia*.* This effect was confirmed in vitro, as ATP-induced secretion of tau-containing exosome was significantly suppressed by GSK1482160 treatment from primary murine microglia, but not from neurons or astrocytes.

**Discussion:**

The oral administration of P2RX7 inhibition mitigates disease phenotypes in P301S mice, likely by suppressing release of microglial exosomes. P2RX7 could be a novel therapeutic target for the early stage tauopathy development.

## Background

Neurofibrillary tangles, primarily composed of intracellular aggregates of hyperphosphorylated microtubule-associated protein tau (tau), are a pathological hallmark in Alzheimer’s disease (AD) and known to progress from the entorhinal cortex (EC) to the hippocampal regions in the early stage of AD. The precise mechanism underpinning this spread is yet to be elucidated, and there has been no successful drug to halt tau pathology progression in the early stages of AD.

Microglia, the innate immune cells in the central nervous system (CNS), play a pivotal role in spreading tau by releasing exosomes, small extracellular vesicles (EVs: 50–150 nm), which contain and transfer pathological tau in the tauopathy mouse model [1]. Other groups demonstrated that exosomes isolated from a different tau mouse model can spread tau pathology after intracranial injection into the recipient mice [2] and AD-associated *BIN1* is critical in microglia-mediated spread of tau pathology via exosome secretion [3]. These evidences support the idea that targeting specific molecules to suppress exosome secretion from microglia may lead to halt tau propagation. Among many possible molecules regulating exosome release, we focused on P2X purinoceptor 7 (P2RX7), an ATP-evoked Na^+^/Ca^2+^ channel predominantly expressed in microglia [4]. Purinergic receptors have important multifaceted properties in the CNS; they not only contribute to neurotransmission and neuromodulation but also to the ATP-driven chemotactic response [5, 6]. Previous studies have shown increased expression of P2RX7 near amyloid-β (Aβ) plaques in the brains of AD patients and AD animal models [7, 8], suggesting a role of P2RX7 in disease progression. Activation of P2RX7 has been shown to trigger Na^+^/Ca^2+^ influx, plasma membrane depolarization, secretion of EVs, chemotaxis and activation of inflammasomes [9]. Pharmacologic inhibition or genetic deletion of *P2rx7* in rodents could alter the exosome release from macrophages and other myeloid cells [10–12]. However, the role of P2RX7 has never been examined in the context of tau spread through exosome secretion in tauopathy animal models.

GSK1482160 is an orally applicable and CNS-penetrant selective antagonist of P2RX7, and has been successfully clinically tested in phase I study [13, 14]. Here, we have comprehensively tested the therapeutic effect of GSK1482160 in P301S tau mouse model [15] by behavioral tests, biochemistry and neuropathology. We discovered that GSK1482160 treatment reduced accumulation of misfolded tau and restored cognitive function in P301S mice, likely via suppressing exosome secretion. Furthermore, we validated the predominant effects of GSK1482160 on exosome secretion from microglia in vitro.

## Materials and methods

Note: Methods and any associated references are available in the online version of the paper.

## Results

### Pharmacological blockade of P2RX7 decreases accumulation of misfolded tau aggregates in P301S mouse brain

**Fig. 1:**
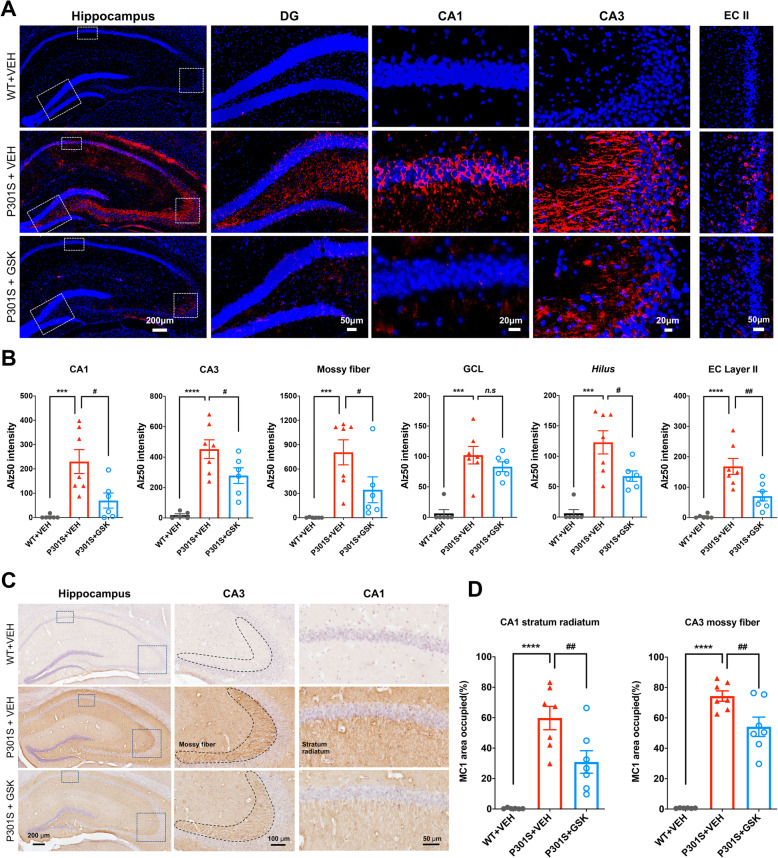
GSK1482160 treatment reduced accumulation of misfolded tau in P301S mouse brain. **a-b**. Alz50 staining (**a**) and quantification (**b**) in the hippocampal and cortical regions of mice ± GSK1482160 treatment. **c-d**. MC1 staining (**c**) and quantification (**d**) in the stratum radiatum of CA1 and mossy fibers of CA3 of the hippocampal regions. **b**, **d**: ^***^p < 0.001 and ^****^p < 0.0001 compared with WT + VEH group; ^#^*p* < 0.05 and ^##^*p* < 0.01 compared with P301S + VEH group; *n.s* denotes no significance as determined by one-way ANOVA (alpha = 0.05) and Dunnett’s *post-hoc*. *n* = (6, 7, 7) mice for WT + VEH, P301S + VEH and P301S + GSK groups. Graphs indicate mean ± s.e.m.

To determine the effect of pharmacological blockade of P2RX7 on tau pathology development in P301S mice, we performed immunohistochemistry (IHC) and biochemical analysis of tau pathology after GSK1482160 or vehicle administration from 3 months of age for 30 days. There was no significant change in body weight during the treatment period among groups (Supplementary Fig. 1A). We first examined the effect of GSK1482160 treatment on tau accumulation in P301S mice. The total human tau levels in the hippocampus and cortex were unchanged between P301S mice with treatments, showing that GSK1482160 treatment has no effect on P301S tau transgene expression (Supplemental Table 1 and 2). Interestingly, pSer199 tau levels were significantly reduced by GSK1482160 treatment in the hippocampal but not the cortical regions of P301S mice (Supplemental Table 1 and 2). In contrast, pT181 and pT231 tau levels were unchanged in both regions of P301S mice between the two treatment groups. Alz50 and MC1 are two of the earliest markers of the misfolded tau [16, 17] and detect AD-specific epitopes formed by two discontinuous portions of tau: _313_VDLSKVTSKC_322_ and _7_EFE_9_ [18, 19]. Importantly, the level of Alz50 and MC1 reactivity correlates with the severity and progression of AD [19]. Alz50 immunoreactivity in vehicle-treated P301S group was significantly elevated in both pyramidal cell bodies and neuropils of the Cornus Ammonis (CA)1, CA3, hilus, granular cell layer (GCL) of dentate gyrus (DG), and entorhinal cortex (EC) layer II compared to wild-type (WT) group (Fig. 1a-b, red columns), consistent with previous findings [20]. Strikingly, GSK1482160 treatment significantly decreased Alz50 immunoreactivity in these regions (Fig. 1a-b, blue columns). We observed similar findings by MC1 staining: MC1^+^ misfolded tau was prominently enriched in the stratum radiatum of CA1 and mossy fibers of CA3 in vehicle-treated P301S group, which was significantly reduced by GSK1482160 treatment (Fig. 1c-d). These data demonstrate that misfolded tau accumulates in the neuronal cell body and dendrites of hippocampal and EC regions of P301S mice at 4.5 months of age, which was suppressed by GSK1482160 administration.

### Pharmacological blockade of P2RX7 reduces Tsg101^+^ immunoreactivity in P301S tau mice

**Fig. 2:**
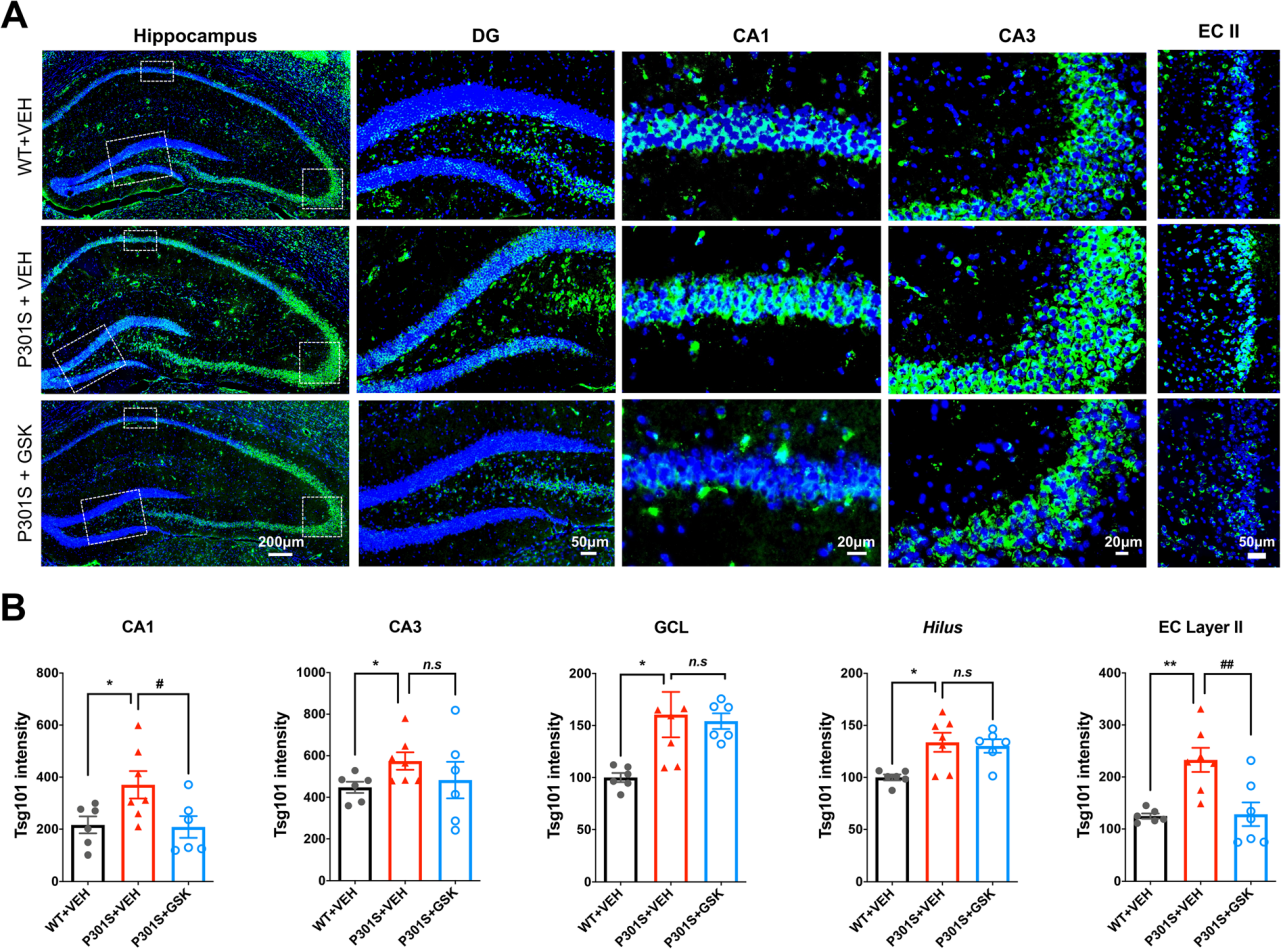
GSK1482160 treatment reduced Tsg101^+^ immunoreactivity in P301S mouse brain. **a**. Tsg101 staining (green) in the hippocampus and cortex of mice ± GSK1482160 treatment, DG: dental gyrus; CA1 and CA3 of hippocampus; EC II: entorhinal cortex layer II. Scale bars = 200, 50, 20, 20 and 50 μm (left to right). **b**. Quantification of Tsg101 fluorescent intensity. ^*^p < 0.05 and ^**^p < 0.01 compared with WT + VEH group; ^#^*p* < 0.05 and ^##^*p* < 0.01 compared with P301S + VEH group; *n.s* denotes no significance as determined by one-way ANOVA (alpha = 0.05) and Dunnett’s *post-hoc*. *n* = (6, 7, 7) mice for WT + VEH, P301S + VEH and P301S + GSK group. Graphs indicate mean ± s.e.m.

Exosomes mediate trans-cellular communication in the CNS, including neuron-microglia interaction in health and disease [21, 22]. To determine how P2RX7 regulates secretion of exosomes, we performed IHC against tumor susceptibility gene 101 (Tsg101), a specific marker of ESCRT-I involved in exosome synthesis. Tsg101 immunoreactivity was higher in the GCL of DG, CA1 and CA3 regions of the hippocampus and EC layer II in vehicle-treated P301S group compared to WT group (Fig. 2a-b). Remarkably, GSK1482160 treatment suppressed Tsg101 accumulation in the CA1, GCL and EC layer II in P301S mice (Fig. 2a-b). The reduction in Tsg101 accumulation was not due to the neuronal cell loss, because there were still comparable numbers of NeuN^+^ neurons in the hippocampal region of P301S mice at this age (Supplementary Fig. 1B). Together, these results show that Tsg101^+^ exosomes or intraluminal vesicles (ILVs) are significantly accumulated in hippocampal and EC neurons in P301S mice, which is partially reversed by GSK1482160 treatment. Concomitant reduction in tau aggregation may suggest the role of exosomes as vehicles of tau between the specific brain regions.

### Association of misfolded tau with Tsg101 in P301S mouse brain

**Fig. 3:**
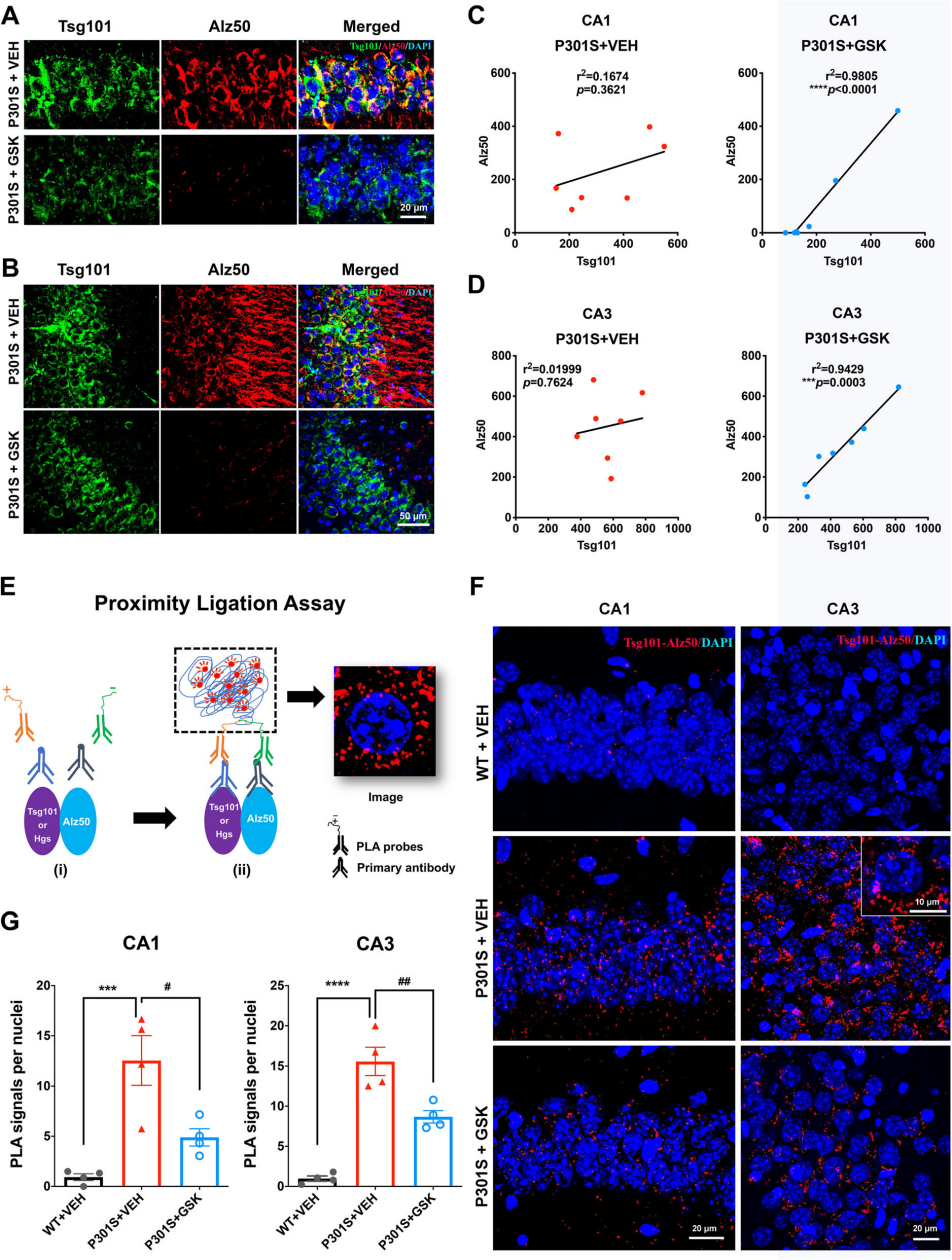
Co-localization and correlation of Alz50 and Tsg101 immunoreactivity in P301S mouse brain. **a**, **b**. Laser-scanning confocal microscopic imaging of Tsg101 and Alz50 staining in the CA1 (**a**) and CA3 (**b**) hippocampal region. **c**, **d**. Scattered plot for the Pearson’s correlation analysis of Alz50 and Tsg101 signal for CA1 (**c**) and CA3 regions (**d**), respectively. Each dot represents an individual animal from P301S + VEH group (red) and P301S + GSK1482160 group (blue). **e**. Schematic diagram of PLA, only occurs when proteins are in close proximity (20–50 nm). **f**. Representative laser-scanning confocal microscopic images of PLA signals between Tsg101 and Alz50 (red) with Dapi (blue) at CA1 and CA3 of the hippocampus. **g**. Quantification of the PLA^+^ immunopuncta for Tsg101-Alz50 at CA1 and CA3 hippocampal regions. ***p <0.001, ^****^p < 0.0001 compared with WT + VEH group; ^#^*p* < 0.05 and ^##^*p* < 0.01, compared with P301S + VEH group; *n.s* denotes no significance as determined by one-way ANOVA (alpha = 0.05) and Dunnett’s *post-hoc*. *n* = (4, 4, 4) mice for WT + VEH, P301S + VEH and P301S + GSK groups. Graphs indicate mean ± s.e.m.

To determine whether these tau aggregates were sorted in exosomal cargo to be migrated in the hippocampal region, we examined the co-localization and correlation of Alz50 and exosome component by laser-scanning confocal microcopy. Exosomal cargo proteins are recruited by the exosome-specific ESCRT-I component Tsg101 [12]. We observed partial co-localization of Alz50 and Tsg101 in the CA1 and CA3 regions in vehicle-treated P301S mice, which is diminished by GSK1482160 treatment (Fig. 3a-b). The scattered plot analysis shows the close association of the amount of Alz50 and Tsg101 in CA1 and CA3 regions unexpectedly only in GSK1482160-treated group (Fig. 3c-d). This suggests that suppression of EV-dependent tau release by the drug treatment contained Alz50^+^ tau in Tsg101^+^ area in these regions (Fig. 3c-d). The lack of association in vehicle-treated group implicates that Alz50^+^ tau signal, although upregulated in CA1 and CA3 regions of this group, are released from EVs after being transferred from microglia in these areas. This approach has some limitation in quantifying the EV-mediated tau transport. Thus, we examined whether Tsg101^+^ immunopuncta is in close proximity to Alz50^+^ misfolded tau using the proximity ligation assay (PLA) representing their close association (close proximity 20–50 nm) [23] (Fig. 3e). We quantified the number of PLA^+^ Tsg101/Alz50 complex in CA1 and CA3 regions, which are significantly reduced by GSK1482160 treatment compared to vehicle-treated P301S mice (Fig. 3f-g). It has been reported that the ESCRT-0 component hepatocyte growth factor-regulated tyrosine kinase substrate (Hgs) is also required for exosome formation and/or secretion [24]. Hgs was also in close proximity to Alz50 as determined by Hgs/Alz50 PLA, which was reduced by GSK1482160 treatment (Supplementary Fig. 2A-B). Taken together, these data indicate that increased number of Alz50^+^ misfolded tau associated with Tsg101^+^ exosomes or ILVs in the hippocampal region, which was suppressed by GSK1482160, suggesting that P2RX7 regulates pathological tau transfer to hippocampal neurons via exosomes.

### Pharmacological blockade of P2RX7 rescues cognitive deficits in Y maze, pre-pulse inhibition and contextual fear conditioning of P301S mice

**Fig. 4:**
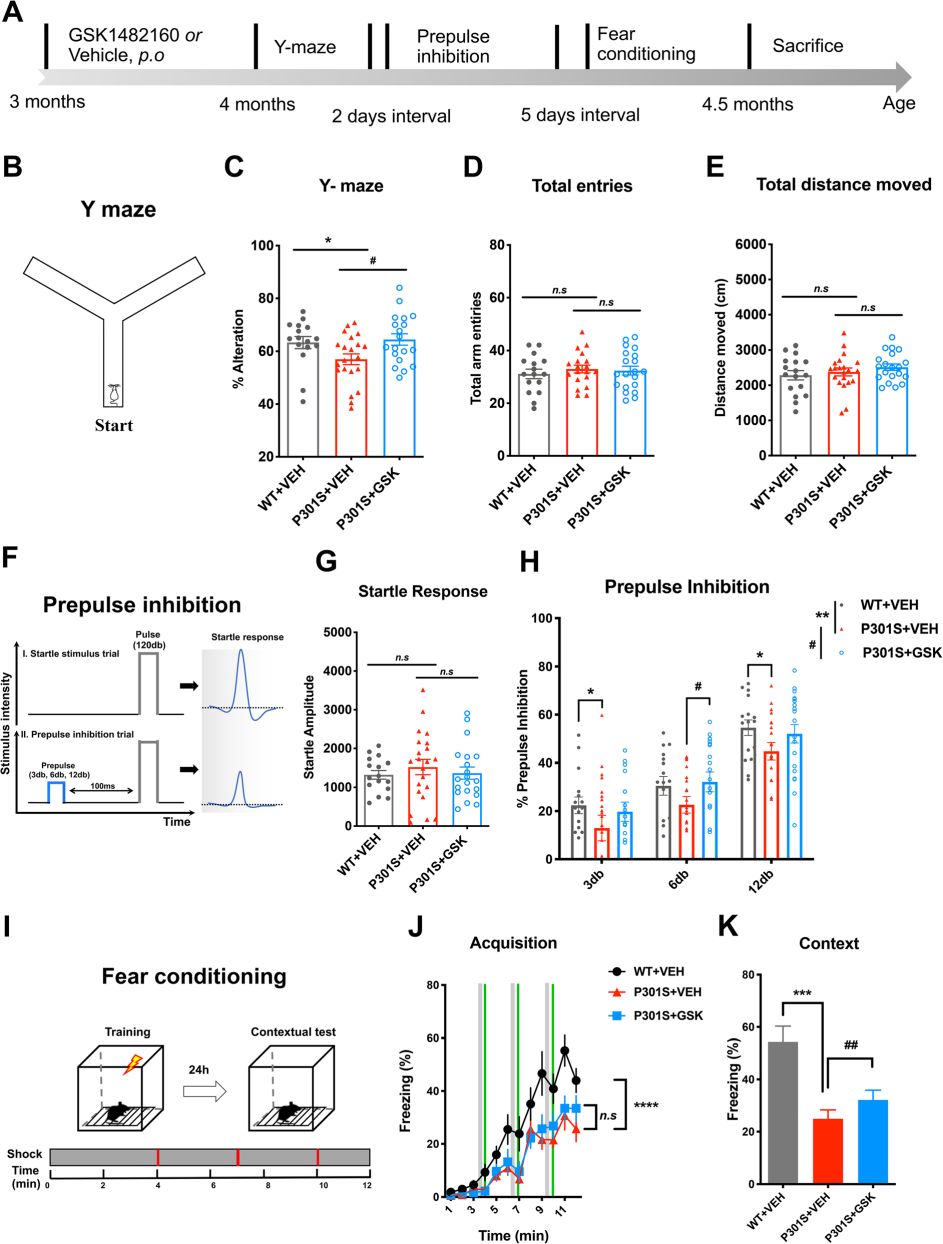
Behavioral testing of P301S mice after GSK1482160 treatment. **a**. Schematic diagram of behavioral tests. **b-e**. Schematic diagram of Y-maze (**b**). Percentage of spontaneous alternation (**c**), number of total arm entries (**d**) and total distance moved during tests (**e**) in Y-maze. ^*^p < 0.05, P301S + VEH vs. WT + VEH; ^#^*p* < 0.05, P301S + GSK vs. P301S + VEH as determined by one-way ANOVA (alpha = 0.05) with Dunnett’s *post-hoc*. n = (17, 21, 20) mice for WT + VEH, P301S + VEH and P301S + GSK groups. Graphs indicate mean ± s.e.m. **f** and **g**. Schematic diagram of pre-pulse inhibition test (**f**). Acoustic startle amplitude as measured in trials with 120 db without a pre-pulse (**g**). PPI (%) at three different pre-pulse intensities (3, 6 and 12 db above 65db background) (H). ^*^p < 0.05, ^**^p < 0.01, P301S + VEH vs. WT + VEH; ^#^*p* < 0.05, P301S + GSK vs. P301S + VEH, *n.s* denotes no significance, as determined by two-way ANOVA (alpha = 0.05) and Bonferroni’s *post-hoc*. n = (17, 21, 20) mice for WT + VEH, P301S + VEH and P301S + GSK groups. **i-k.** Schematic diagram of fear conditioning (**i**). **j.** Percentage of freezing time of each minute during cued fear acquisition. ^****^p < 0.0001, P301S + VEH vs. WT + VEH as determined by two-way ANOVA (alpha = 0.05) and Bonferroni’s *post-hoc*. n = (17, 21, 20) mice for WT, P301S + VEH and P301S + GSK groups. **k.** % freezing during contextual text. ^***^p < 0.001, P301S + VEH vs. WT + VEH; ^##^*p* < 0.01, P301S + GSK vs. P301S + VEH as determined by paired Student’s *t*-test at each minute during the 5 min period. Graphs indicate mean ± s.e.m.

To determine the effect of P2RX7 inhibition on cognitive function, we performed batteries of the behavioral assays with vehicle or GSK1482160-treated P301S mice (Fig. 4a). The Y-maze test was performed to evaluate spatial working memory at 4 months of age (Fig. 4b). Vehicle-treated P301S mice showed a significant decrease in alternations compared to age-matched WT mice, which was rescued by GSK1482160 treatment (Fig. 4c). There was no difference in the total number of entries into each arm and total distance moved among the three groups, suggesting that spontaneous motor activity was unaffected by P301S tau transgene expression or drug treatment at this age (Fig. 4d-e). A pre-pulse inhibition (PPI) test is widely used to measure deficits in information-processing abilities or sensorimotor gating across species, including humans and rodents [25]. There were no significant differences in startle response among groups at baseline (Fig. 4f-g). Interestingly, the % PPI, an index of sensorimotor gating, was significantly lower in P301S mice at 3db and 12 db (Fig. 4h) compared to that in WT mice (main effect *p* = 0.0074) while GSK1482160 treated-P301S mice showed recovery of %PPI at 6db compared to vehicle treated P301S mice (treatment effect *p* = 0.0206). We next performed the fear-conditioning test, assessing the spatial memory ability in WT and P301S mice with or without GSK1482160 treatment (Fig. 4i). We observed significant deficits in fear memory acquisition in vehicle treated P301S compared to WT mice, which was not recovered by GSK1482160 treatment (Fig. 4j). We then tested their contextual fear memory 24 h later, which is largely dependent on hippocampal function. Interestingly, we observed significantly higher contextual freezing in GSK1482160 treated P301S mice compared to that of vehicle treated P301S mice (Fig. 4k), suggesting treatment effect of P2RX7 inhibition on hippocampal memory. These results demonstrate that GSK1482160 treatment on P301S mice ameliorates impairment in working memory in Y-maze, information-processing ability in PPI tests and contextual memory in fear-conditioning test.

### GSK1482160 administration induces exosome accumulation in microglia in P301S mice

**Fig. 5:**
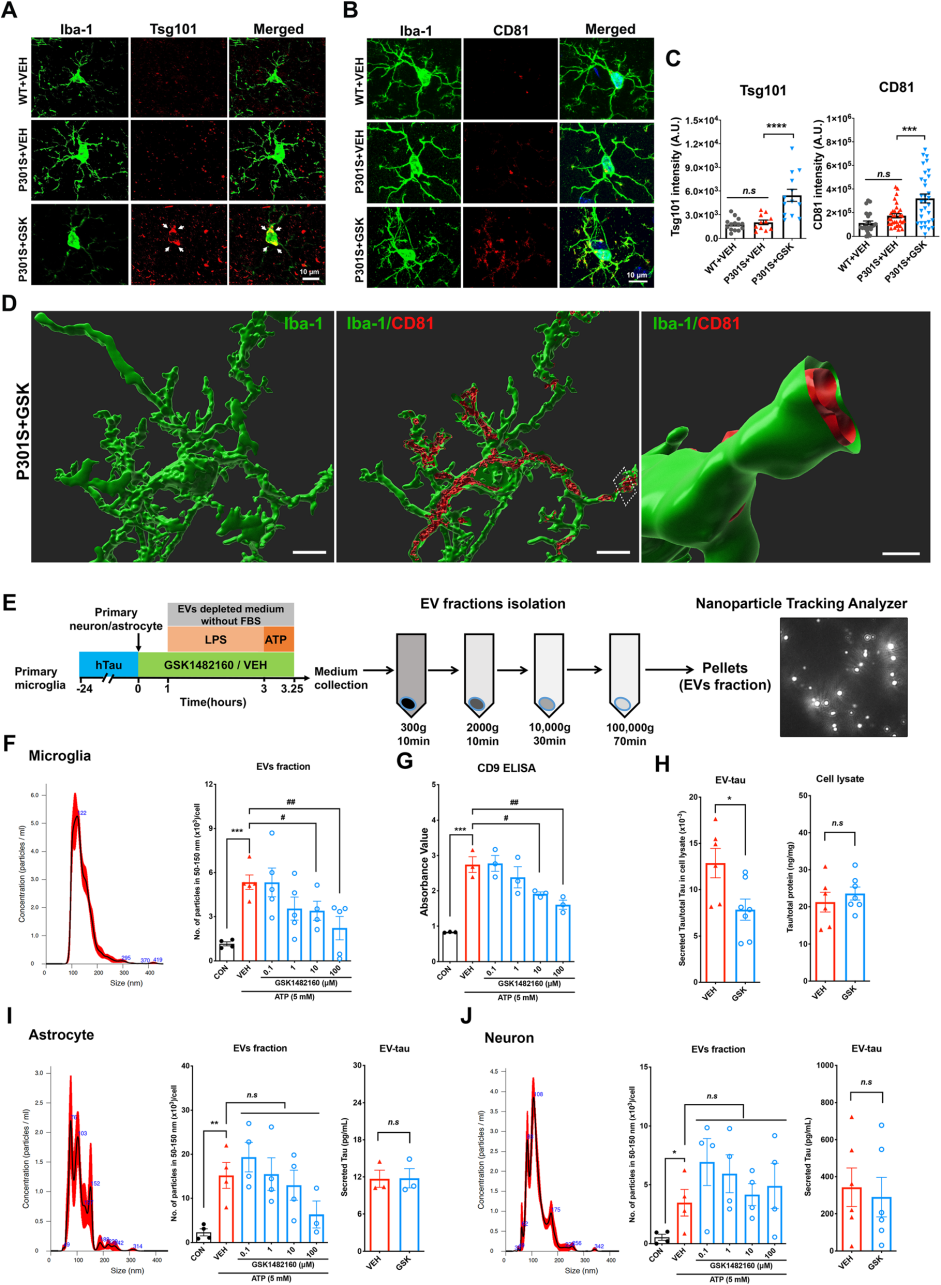
GSK1482160 treatment induced exosomal accumulation in microglia in P301S mice in vivo and suppressed microglial EV secretion in vitro. **a-b.** Immunofluorescence images of mouse hippocampal CA1 regions captured by the laser-scanning confocal microscopy. Iba-1 (green) co-stained with Tsg101 (red, left panel) and CD81 (red, right panel). **c.** Quantitative analysis of Tsg101 and CD81 florescent intensity in Iba-1^+^ positive cells**:** ^***^p < 0.001, ^****^p < 0.0001, P301S + GSK compared with P301S + VEH group, *n.s* denotes no significance as determined by one-way ANOVA (alpha = 0.05) and Dunnett’s *post-hoc*. Each dot represents an individual cell, Tsg101: n = (15, 13, 13) and CD81: n = (25, 32, 32) for (WT + VEH, P301S + VEH, P301S + GSK1482160) from 5 to 7 animals per group. Graphs indicate mean ± s.e.m. **d.** Re-constructed structure of exosome in microglia by Imaris software from P301S + GSK1482160 group. Membrane bound CD81(red) is shown in microglial processes (green). Scale bar 5, 5 and 2 μm. **e.** Scheme of EV isolation protocol from primary culture microglia, astrocyte and neuron conditioned media, followed by NTA of isolated EVs. Microglia were stimulated by LPS and ATP. All of the cells were pretreated by GSK1482160 or vehicle. **f-h.** EVs were isolated from the culture media of primary microglia without stimulation of ATP (control) or stimulated by 5 mM ATP and treated by either vehicle or GSK1482160 (0.1-100 μM). NTA plot of mode size and concentration (**F left**), particle numbers (**f** right), and CD9 ELISA (**g**) of microglial EVs. Total tau ELISA for the EV fraction and cell lysate from microglia pretreated with the vehicle or 10 μM of GSK1482160 (**h**). **i**-**j.** NTA and tau ELISA of isolated EVs from primary astrocyte (**i**) and neuron (**j**). ^*^p < 0.05, ^**^p < 0.01 and ^***^p < 0.001 compared with Control group; ^#^*p* < 0.05 and ^##^*p* < 0.01 compared with ATP-treated group; *n.s* denotes no significance as determined by one-way ANOVA (alpha = 0.05) and Dunnett’s *post-hoc*; Student’s *t*-test for the total tau ELISA. Data are representative of at least three independent experiments. Graphs indicate mean ± s.e.m.

We previously reported that depletion of microglia or inhibition of exosome synthesis suppressed accumulation of tau in the hippocampus in this mouse model [1]. Thus, the reduction of tau pathology and recovery of cognitive function by GSK1482160 treatment on P301S mice could be mediated by suppressing microglial exosome secretion. To determine the effect of GSK1482160 treatment on microglial exosome efflux, we performed immunofluorescence against Iba-1 and Tsg101 or CD81, another exosomal marker and tetraspanin superfamily member predominantly expressed on the plasma membrane (Fig. 5a-b). Interestingly, we found significantly increased signal of Tsg101 in microglia in GSK1482160-treated P301S mice compared to the vehicle-treated P301S mice, suggesting that GSK1482160 treatment accumulated Tsg101^+^ ILVs by inhibition of their secretion from microglia (Fig. 5a and c left). There was no difference in Tsg101 immunoreactivity in microglia between the vehicle-treated P301S and WT groups. In contrast to this finding in microglia, Tsg101 immunoreactivity in astrocytes, oligodendrocytes or neurons was not enhanced by GSK1482160 treatment (Supplemental Fig. 3A-C). Furthermore, immunoreactivity of CD81 was also increased in microglia in the mouse brain treated by the GSK1482160 (Fig. 5b and c right). Following the assessment of 3D surface rendering reconstruction images of CD81 in microglia by Imaris software, most of CD81 signal was bound to the inner plasma membrane of microglial processes (Fig. 5d), suggesting the retention of ILVs by GSK1482160. In order to determine whether GSK1482160 treatment affects the microglia phenotype and cytokine secretion profile, we performed microglia morphology analysis and examined the expression level of *Il6*, *Il10*, *Tgfb1*, *Il1a*, *Il1b*, *Il18*, *Ifng* and *Tnfa* by qRT-PCR. Immunofluorescence against Iba-1 and purinergic receptor P2Y12 (P2ry12), a specific and representative marker of resident microglia, did not show a change in microglia population among 3 groups (Supplementary Fig. 4A-B). Microglial morphology analysis using Iba-1 stained images and Imaris software revealed that P301S mice significantly had longer processes and higher branches with more intersections compared to WT, which change was not normalized by GSK1482160 treatment (Supplementary Fig. 4 C-E). The production of pro- and anti-inflammatory cytokines were not changed among 3 groups except 3-fold higher expression level of *Tnfa* in P301S mouse*,* which was not normalized by GSK1482160 treatment (Supplemental Fig. 4F). Finally, we evaluated if GSK1482160 treatment affect microglial phagocytic function by immunofluorescence against CD68, a marker for phagocytic myeloid cells. Although there was an increase in the number of CD68^+^ microglia in P301S mice compared to WT mice, GSK1482160 treatment did not show any effect (Supplemental Fig. 5A-C). This is consistent with a previous study showing that P2X7R deficiency did not modify the microglial numbers, cytokine secretion profile, or phagocytic activity while it reduced Aβ deposition, rescued cognitive deficits and improved synaptic plasticity in APPPS1 mice [26]. Together, these results suggested that the effect of GSK1482160 on tau pathology in P301S mice is mediated by suppressed exosome secretion from microglia, which is independent of microglial morphology or phagocytic function.

### Pharmacological blockade of P2RX7 suppresses EV secretion by microglia in vitro

P2RX7 is predominantly present in microglia in central nervous system, although a limited amount may still be expressed in neuron and astrocyte [27]. To determine which cell type is mainly affected by GSK1482160 for secretion of tau and exosomes, we tested primary cultured microglia, neurons and astrocytes followed by GSK1482160 treatment as previously described [1]. Microglia were isolated from WT P0 pups and pre-incubated with aggregated human tau for 24 h before GSK1482160 treatment ranging from 0.1–100 μM and induction of exosome secretion by LPS and ATP (Fig. 5e). Since P301S mice express mutant tau mostly in neurons followed by astrocytes and not in microglia [28], and our previous study show very limited amount of hTau phagocytosis by these cell types [1], we examined the effect of GSK1482160 treatment on hTau secretion from neurons and astrocytes isolated from E16 P301S embryonic brains. The cells were treated with ATP, and the EV-enriched fractions were isolated from the ATP-treated conditioned media by sequential centrifugations. Nanoparticle tracking analysis (NTA) revealed that ATP stimulation significantly increased EV secretion from microglia in the exosomal range (50–150 nm) (Fig. 5f). Notably, pre-treatment with GSK1482160 significantly suppressed exosome secretion from microglia in a dose-dependent manner (Fig. 5f). We also obtained similar results using microglial cell line BV-2 (Supplementary Fig. 6A-C). The dose-dependent suppression of exosome secretion by GSK1482160 treatment was further confirmed by ELISA-based quantification of exosome-specific marker CD9 (Fig. 5g). In accord, hTau level in EVs secreted from hTau-phagocytosed and ATP-stimulated microglia was significantly reduced by GSK1482160 pre-treatment at 10 μM (Fig. 5h left). There was no change in hTau in microglia cell lysates by the GSK1482160 pre-treatment (Fig. 5h right). EVs and tau secretion from P301S mouse-derived neurons and astrocytes were also tested in the same manner. Although ATP stimulation significantly increased EV secretion from neurons or astrocytes, there was no significant effect of GSK1482160 pre-treatment on EV secretion from either cell types (Fig. 5i-j). Tau secretion was detected in the EV of ATP-stimulated neurons and astrocytes to a lesser extent comparing to the amount of Tau (close to 1500 pg/mL) secreted from microglia, but these were not inhibited by GSK1482160 pre-treatment (Fig. 5i-j). These data suggest that EV secretion is inducible from neurons or astrocytes by ATP stimulation, but it is insensitive to P2RX7 inhibition. Together, these results indicate that GSK1482160 significantly suppresses exosome and tau secretion from hTau-phagocytosed microglia but not from neurons or astrocytes.

## Discussion

**Fig. 6:**
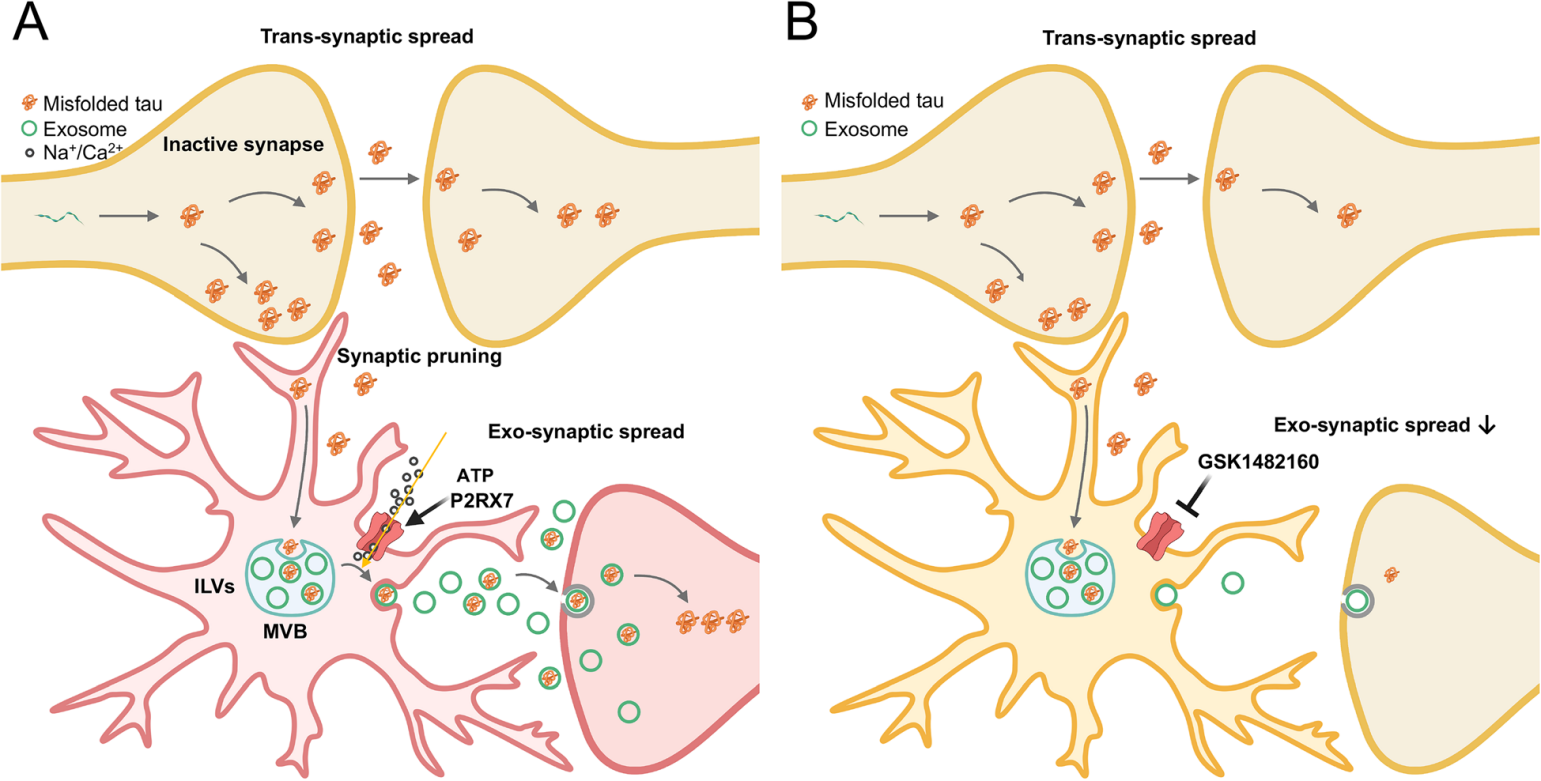
Scheme of the potential mechanisms underlying the reduction of tau pathology via targeting microglial P2RX7 in tau transgenic mouse model. **a**. Tau aggregates may spread trans-synaptically if the synapses are intact. However, in case of tau accumulation, synapses are inactivated and subjected to microglial sensing and pruning in disease conditions. Phagocytosed synapses contain tau aggregates, which will be transported through endosomal pathways, and many of them will be digested through the endolysosomal pathway. In parallel, tau aggregates in the late endosome will be incorporated into the ILVs of MVBs, which will be secreted as exosomes and propagated into nearby neurons. P2RX7 mediates secretion of both exosomes and microvesicles. **b**. Inhibition of P2RX7, mainly expressed in microglia, reduces secretion of EVs and potentially enhances intracellular clearance of tau through endolysosomal degradation, suppressing the microglia-mediated tau propagation in P301S mice

In the present study, we have demonstrated that pharmacologic blockade of P2RX7 at an early stage in P301S mice significantly decreased tau accumulation in the hippocampal and EC regions and ameliorated hippocampal memory deficits. Meanwhile, there was strong correlation between the accumulation of exosomal marker Tsg101 and the misfolded tau marker (Alz50) in the hippocampal region of P301S mice. In addition, the ATP-induced secretion of exosomes from murine microglia was significantly suppressed by the P2RX7 inhibitor, whereas it had no effect on tau or EV secretion from primary murine neurons or astrocytes in vitro*.* Taken together, these findings establish a functional role of P2RX7 on microglial exosome secretion in tau pathology progression, and would serve as promising preclinical data for the development of P2RX7 inhibitors as potential therapeutics of AD (summarized in Fig. 6). Moreover, P2RX7 deficiency decreased Aβ load and also rescued cognitive deficits in APP/PS1 mice [26]. Thus, P2RX7 may be a beneficial target for suppressing both Aβ accumulation and tauopathy development in AD.

The P301S mouse model recapitulates not only tau pathology but also neurological deficits of human tauopathies with dementia including AD at the early stage [15]. Moreover, behavioral abnormalities shown by Y-maze, PPI and contextual fear conditioning tests have widely been used to recapitulate human cognitive disorders found in AD, especially hippocampal spatial learning disturbances [29–31]. In the present study, we found spatial learning deficits in P301S mice and its rescue by GSK1482160 treatment. Although behavior deficits in PPI test is a common finding in AD mouse models including rTg4510, N279K and R406W tau models [32–34], a study showed enhanced PPI in P301S mice relative to WT controls at 6 months of age [15]. This could be due to the strain background differences which could affect the behavior of the animals. While they used mice with a C57BL/6 J background, ours were from the B6C3H/F1 background, and will require future experiments for validation.

Although P2RX7 is predominantly expressed in microglia, it is also expressed in oligodendrocytes in the CNS [4] and in astrocytes under inflammatory conditions in P301S mice [35]. Several groups reported the presence of tau in neuron-derived exosomes isolated from the blood and cerebrospinal fluid (CSF) in AD patients [36]. Thus, reduction of tau aggregates and Tsg101 immunoreactivity by GSK1482160 may also reflect the direct effect of P2RX7 inhibition on different cell types expressing P2RX7 in vivo [37]. P2RX7 inhibition did not increase Tsg101 expression in neurons, astrocytes or oligodendrocytes but exclusively in microglia in vivo and in vitro, therefore microglia may be the major source of Tsg101 bound aggregated tau. To determine if P2RX7 is involved in tau-containing EV secretion from these cells, cell type-specific deletion of P2RX7 in tau propagation mouse models would be helpful as a future study.

In addition to exosomes, larger EVs (ectosomes/microvesicles) may also contain abnormally phosphorylated tau [38]. ATP-induced P2RX7 stimulation induced shedding of ectosomes/microvesicles as well as secretion of exosomes [39, 40]. Further investigation is needed to determine the involvement of larger EVs to understand their roles on tau transfer and AD pathobiology. In terms of tau pathology, we found that the hippocampal CA1 region was the most sensitive to a reduction of tau aggregates in the hippocampal region by GSK1482160, which was correlated with a reduction in Tsg101 immunoreactivity. Physical proximity of tau and exosomes was further confirmed by PLA between Alz50 and Tsg101 or Hgs, the ESCRT-0 molecule. These data suggest that exosomal tau transfer may occur in a region-specific manner in P301S mice.

Trans-neuronal propagation of tau aggregates between anatomically connected brain regions was first reported by Braak [41]. Free form tau can be transferred as a monomer or oligomers by endocytosis or macropinocytosis and induce templated conformational changes in the recipient endogenous tau [42]. Recent studies indicate low density lipoprotein receptor-like proteins or heparan sulfate proteoglycan as the molecular mechanisms by which tau protein is transferred [42, 43]. While tau is the principle molecular component in tau pathology, there are considerable evidences to support the roles of extrinsic factors in tau transfer. We have previously reported that EV-associated tau secreted from microglia play a critical role on tau propagation in AAV tau propagation- and P301S mouse model [1]. Our recent study revealed the inflammatory alternation of EV cargo composition released from human primary cultured astrocytes: IL-1β stimulation enhances the EV cargo load of integrins, which facilitate their uptake by neurons, suggesting the high transmissibility of glial EVs under inflammatory conditions [44]. BIN1, the second most significant AD genome-wide association study (GWAS) gene, is present in EVs in the AD CSF, forms complexes with tau and enhances EV secretion [3]. Strikingly, microglia-specific deletion of Bin1 suppresses tau propagation in P301S mice [3]. Conversely, virus-mediated over-expression of BIN1 accelerated tau pathology in P301S mouse brains [45]. These studies suggest the potential contribution of the AD GWAS genes on EV-mediated tau propagation.

## Conclusions

In summary, our study demonstrated that pharmacological block of P2RX7 in P301S mice significantly decreased tau accumulation in the hippocampal and EC regions, and ameliorated hippocampal memory deficits as determined by several behavioral tests. The misfolded tau marker shows co-localization with exosome markers in the affected brain regions, and our in vitro study shows cell type-specific suppression of exosome release by the P2RX7 inhibitor. These findings shed light on the specific P2RX7 blockers as novel disease-modifying agents for AD.

## Supplementary information

**Additional file 1: Supplementary Fig. S1**. No difference in body weight and neuron number after GSK1482160 administration. A. Body weight of animals during the period of oral gavage of GSK1482160 (GSK) or vehicle (VEH) twice per day. B. Neuron-specific expression of NeuN staining in the hippocampus of P301S with GSK1482160 or vehicle treatment. There was no change in the neuron number between two groups. Original images were captured at mouse brain hippocampus by using 20× objective.

**Additional file 2: Supplementary Fig. S2**. PLA signals between Alz50 and Tsg/Hgs in hippocampus. A. Epi-fluorescence microscopic images of Tsg101 and Alz50 staining in the DG, CA1 and CA3 of hippocampal region of P301S mice ± GSK1482160 treatment and wild-type mice. Original images were captured at 20× objective. B. Representative laser-scanning confocal microscopic images of PLA signals between Hgs and Alz50 at CA1 and CA3 of hippocampus (red) with Dapi counterstaining for nucleus (blue). Original images were captured at 63× oil immersion objective.

**Additional file 3: Supplementary Fig. S3**. Tsg101 immunoreactivity in astrocyte, oligodendrocytes, or neurons in CNS with or without GSK1482160 treatment. A. Astrocyte (GFAP, green) co-stained with Tsg101 (red). B. Oligodendrocyte (MOG, green) co-stained with Tsg101 (red). C. Neuron (MAP 2, green) co-stained with Tsg101(red). 4 animals were stained per group. Original images were captured at CA1 region of mouse brain hippocampal field by using 40× oil immersion objective (Leica SP8 Lightning confocal microscopy).

**Additional file 4: Supplementary Fig. S4**. GSK1482160 has no effect on microglia morphology and inflammatory cytokine production. A-B. Microglia stained with Iba-1 (gray scale) and P2ry12 antibodies (green, A) and their quantification (B). Each dot represents an individual animal, 5–6 animals per group. *n.s* denotes no significance as determined by one-way ANOVA (alpha = 0.05) and Dunnett’s post hoc. Graphs indicate mean ± s.e.m. C-E: Morphological analysis of microglial processes stained with Iba-1 by Imaris software. Representative image of microglial process (C), quantification of average process length and branch points (D), ^***^p < 0.001, one-way ANOVA with Dunnett’s post hoc, and Sholl intersections analysis (E), ^****^p < 0.0001, two-way ANOVA with Bonferroni’s post hoc; each dot represents an individual cell, n = (22, 35, 38) for (WT + VEH, P301S + VEH, P301S + GSK1482160) from 5 animals per group. F. The mRNA expression level of pro- and anti-inflammatory cytokine among three groups. ^*^p < 0.05, one-way ANOVA with Dunnett’s post hoc, 5–6 animals per group.

**Additional file 5: Supplementary Fig. S5**. Effect of GSK1482160 on tau pathology was independent of phagocytosis activity of microglia. A and B. Microglia phagocytic function analysis. Microglia (Iba-1, green) co-stained with a lysosome marker CD68 (red) (A); Quantification of the %CD68^+^ area in Iba-1^+^ microglia (B), ^**^p < 0.01, one-way ANOVA with Dunnett’s post hoc, each dot represents an individual cell, n = (61, 59, 61) for (WT + VEH, P301S + VEH, P301S + GSK1482160) from 5 animals per group. C. 3D surface rendering of CD68^+^ area in microglia from P301S + GSK142160 group by Imaris software.

**Additional file 6: Supplementary Fig. S6**. GSK148620 treatment suppress exosomal secretion from murine microglial-like BV2 cells. A. Scheme of EV isolation protocol from murine microglial cell line BV-2 conditioned media after ATP stimulation. B-C. NTA analysis of isolated EVs. ^***^p < 0.001, compared with Control group; ^#^*p* < 0.05, ^##^*p* < 0.01 and ^###^*p* < 0.01, compared with Vehicle-treated group; *n.s* denotes no significance as determined by one-way ANOVA (alpha = 0.05) and Dunnett’s *post-hoc*. Graphs indicate mean ± s.e.m.

**Additional file 7: Supplementary Table 1**. GSK1482160 did not change the total Tau expression in P301S mouse brain hippocampus, but slightly reduced the hyperphosphorylation level of Tau. **Supplementary Table 2**. GSK1482160 did not change the total Tau expression and hyperphosphorylation level in P301S mouse cerebral cortex.

**Additional file 8.** Supplementary Methods Materials and Methods.

## Data Availability

Not applicable.

## Abbreviations

AD: Alzheimer’s disease
CNS: Central nervous system
CSF: Cerebrospinal fluid
CA: Cornus Ammonis
DG: Dentate gyrus
EC: Entorhinal cortex
EV: Extracellular vesicle
GWAS: Genome-wide association study
GCL: Granular cell layer
Hgs: Hepatocyte growth factor-regulated tyrosine kinase substrate
IHC: Immunohistochemistry
ILV: Intraluminal vesicle
tau: Microtubule-associated protein tau
NTA: Nanoparticle tracking analysis
P2RX7: P2X purinoceptor 7
PPI: Pre-pulse inhibition
PLA: Proximity ligation assay
Tsg101: Tumor susceptibility gene 101
WT: Wide-type
