# Supplementary figures and images for: P2RX7 inhibitor suppresses exosome secretion and disease phenotype in P301S tau transgenic mice
Source: Mol Neurodegener. 2020 Aug 18;15:47. doi: 10.1186/s13024-020-00396-2 (PMC7436984; doi:10.1186/s13024-020-00396-2)

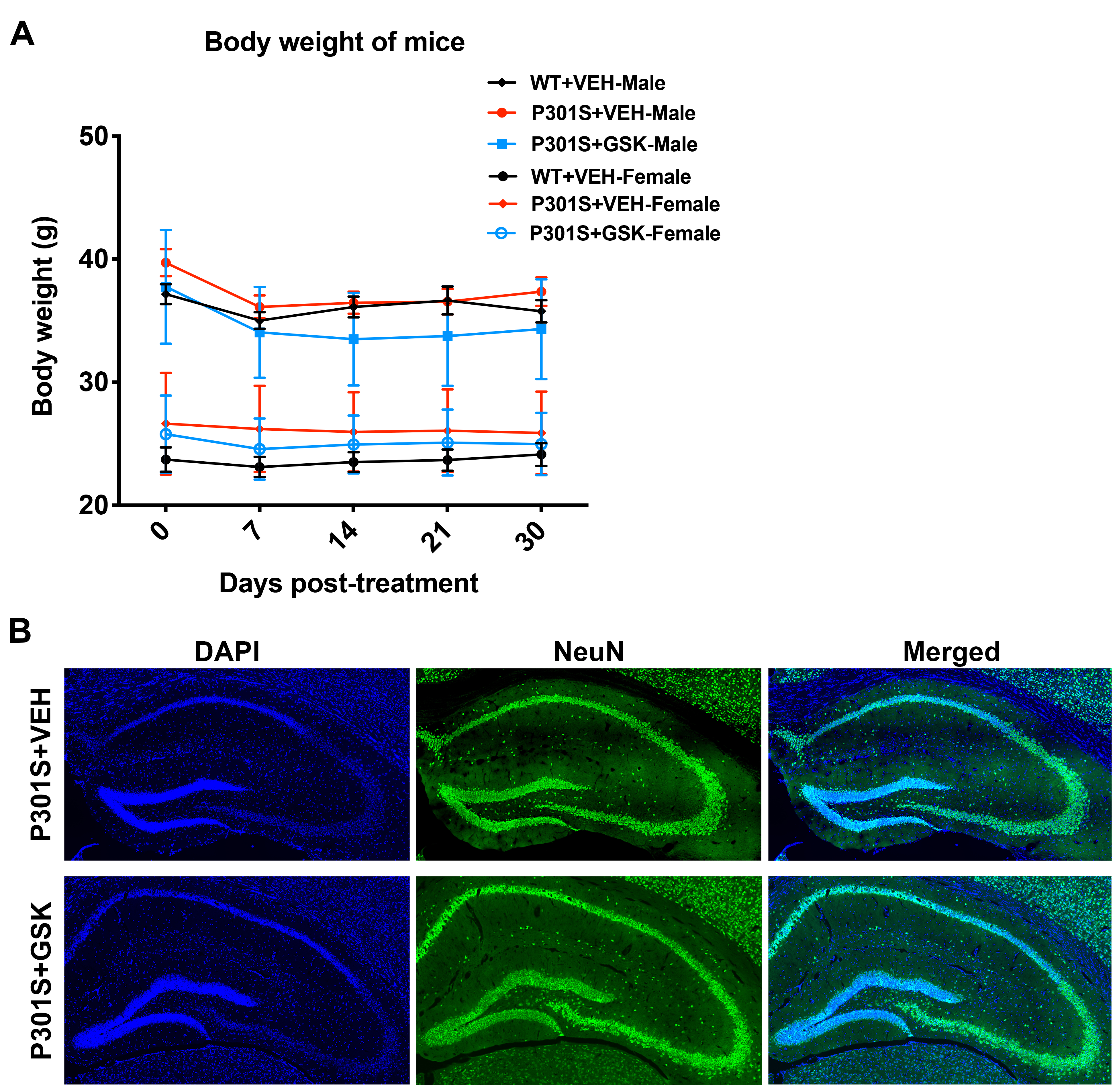

Supplement: Supplementary file 1 — Additional file 1: Supplementary Fig. S1. No difference in body weight and neuron number after GSK1482160 administration. A. Body weight of animals during the period of oral gavage of GSK1482160 (GSK) or vehicle (VEH) twice per day. B. Neuron-specific expression of NeuN staining in the hippocampus of P301S with GSK1482160 or vehicle treatment. There was no change in the neuron number between two groups. Original images were captured at mouse brain hippocampus by using 20× objective. [file 13024_2020_396_MOESM1_ESM.tif]

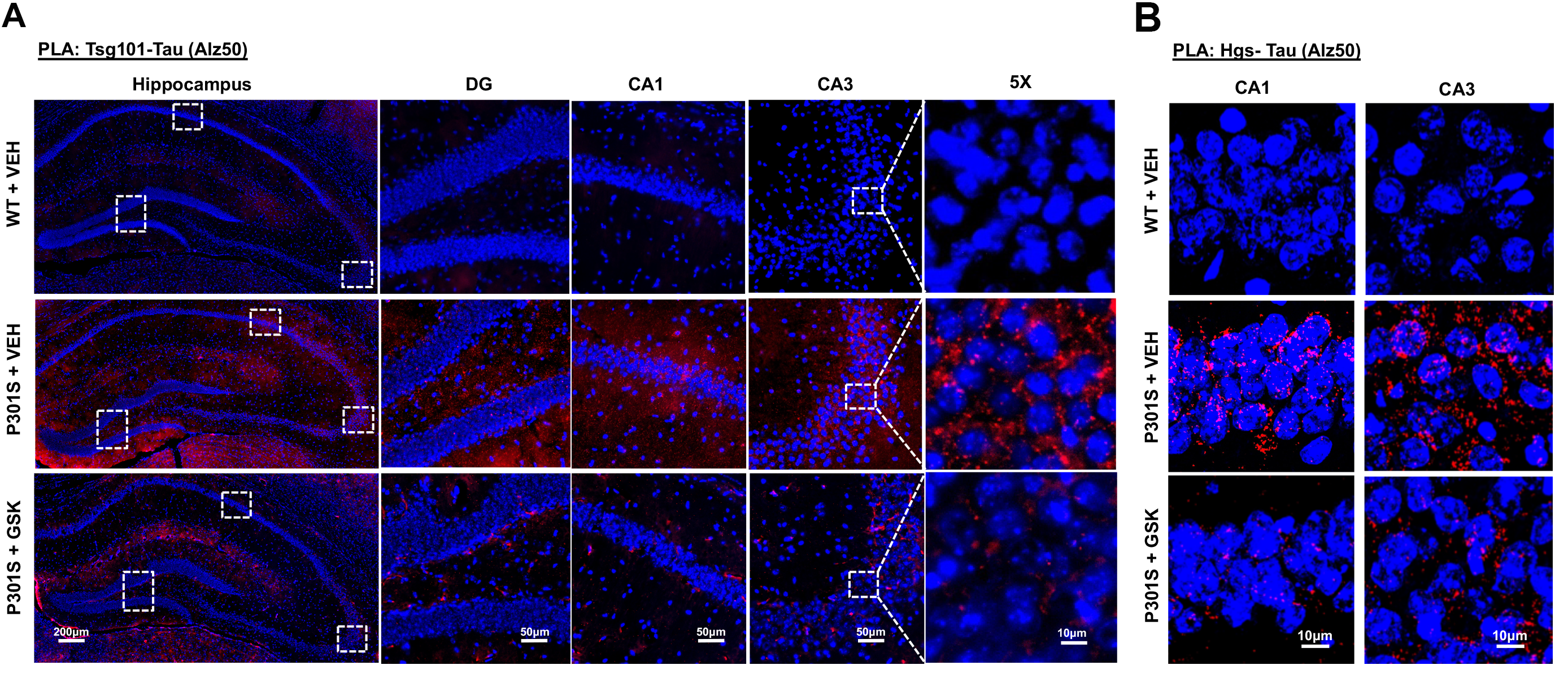

Supplement: Supplementary file 2 — Additional file 2: Supplementary Fig. S2. PLA signals between Alz50 and Tsg/Hgs in hippocampus. A. Epi-fluorescence microscopic images of Tsg101 and Alz50 staining in the DG, CA1 and CA3 of hippocampal region of P301S mice ± GSK1482160 treatment and wild-type mice. Original images were captured at 20× objective. B. Representative laser-scanning confocal microscopic images of PLA signals between Hgs and Alz50 at CA1 and CA3 of hippocampus (red) with Dapi counterstaining for nucleus (blue). Original images were captured at 63× oil immersion objective. [file 13024_2020_396_MOESM2_ESM.tif]

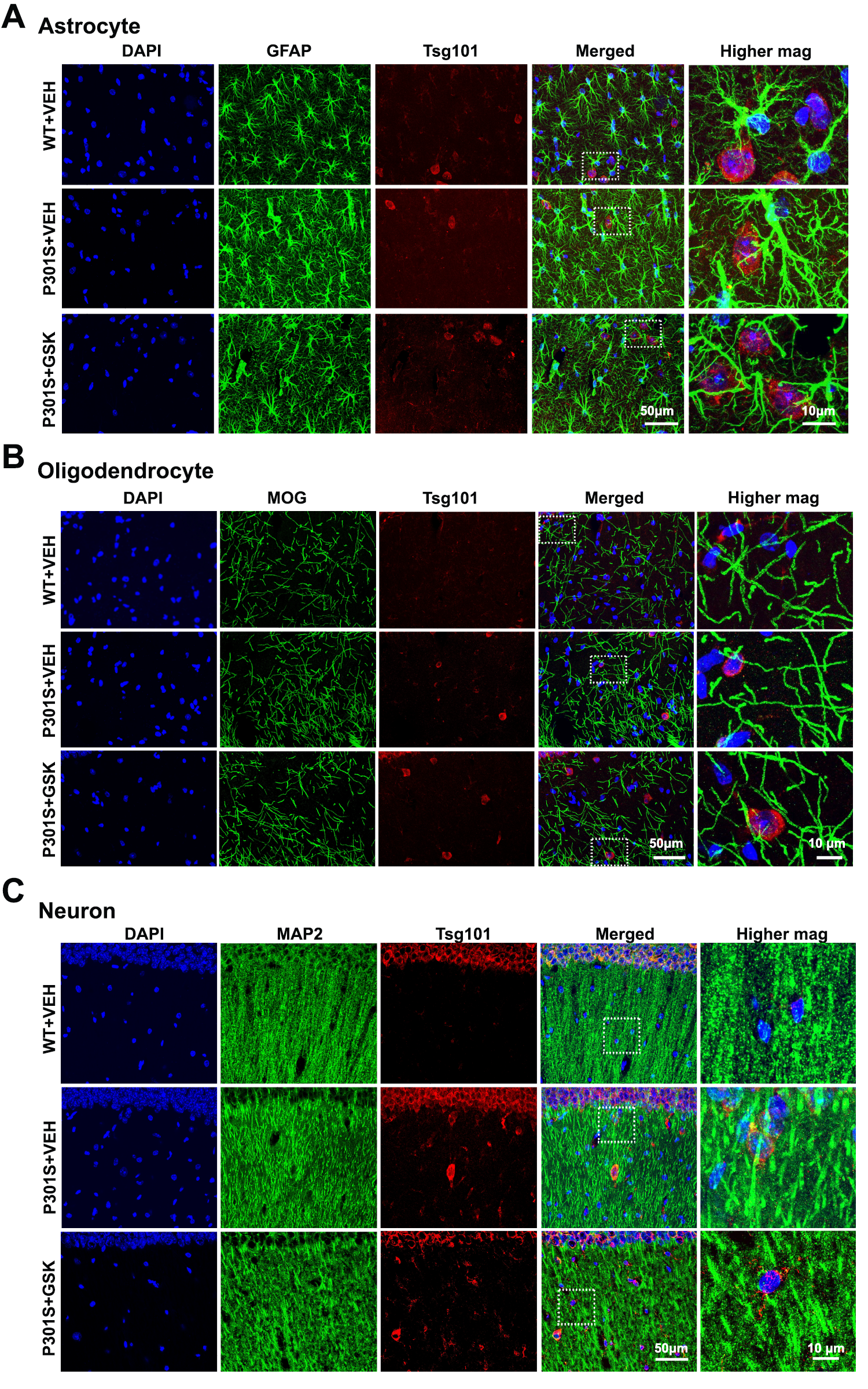

Supplement: Supplementary file 3 — Additional file 3: Supplementary Fig. S3. Tsg101 immunoreactivity in astrocyte, oligodendrocytes, or neurons in CNS with or without GSK1482160 treatment. A. Astrocyte (GFAP, green) co-stained with Tsg101 (red). B. Oligodendrocyte (MOG, green) co-stained with Tsg101 (red). C. Neuron (MAP 2, green) co-stained with Tsg101(red). 4 animals were stained per group. Original images were captured at CA1 region of mouse brain hippocampal field by using 40× oil immersion objective (Leica SP8 Lightning confocal microscopy). [file 13024_2020_396_MOESM3_ESM.tif]

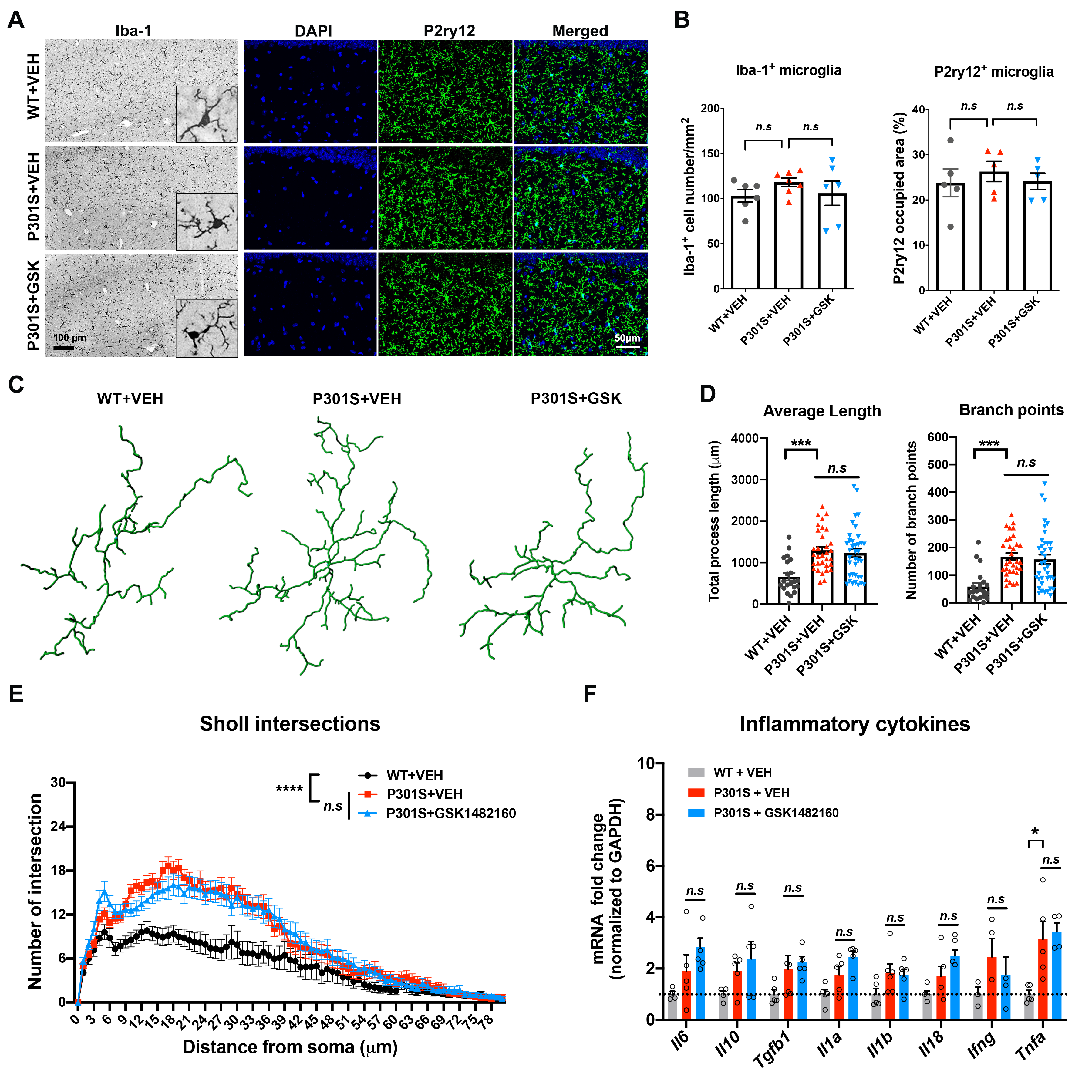

Supplement: Supplementary file 4 — Additional file 4: Supplementary Fig. S4. GSK1482160 has no effect on microglia morphology and inflammatory cytokine production. A-B. Microglia stained with Iba-1 (gray scale) and P2ry12 antibodies (green, A) and their quantification (B). Each dot represents an individual animal, 5–6 animals per group. n.s denotes no significance as determined by one-way ANOVA (alpha = 0.05) and Dunnett’s post hoc. Graphs indicate mean ± s.e.m. C-E: Morphological analysis of microglial processes stained with Iba-1 by Imaris software. Representative image of microglial process (C), quantification of average process length and branch points (D), ***p < 0.001, one-way ANOVA with Dunnett’s post hoc, and Sholl intersections analysis (E), ****p < 0.0001, two-way ANOVA with Bonferroni’s post hoc; each dot represents an individual cell, n = (22, 35, 38) for (WT + VEH, P301S + VEH, P301S + GSK1482160) from 5 animals per group. F. The mRNA expression level of pro- and anti-inflammatory cytokine among three groups. *p < 0.05, one-way ANOVA with Dunnett’s post hoc, 5–6 animals per group. [file 13024_2020_396_MOESM4_ESM.tif]

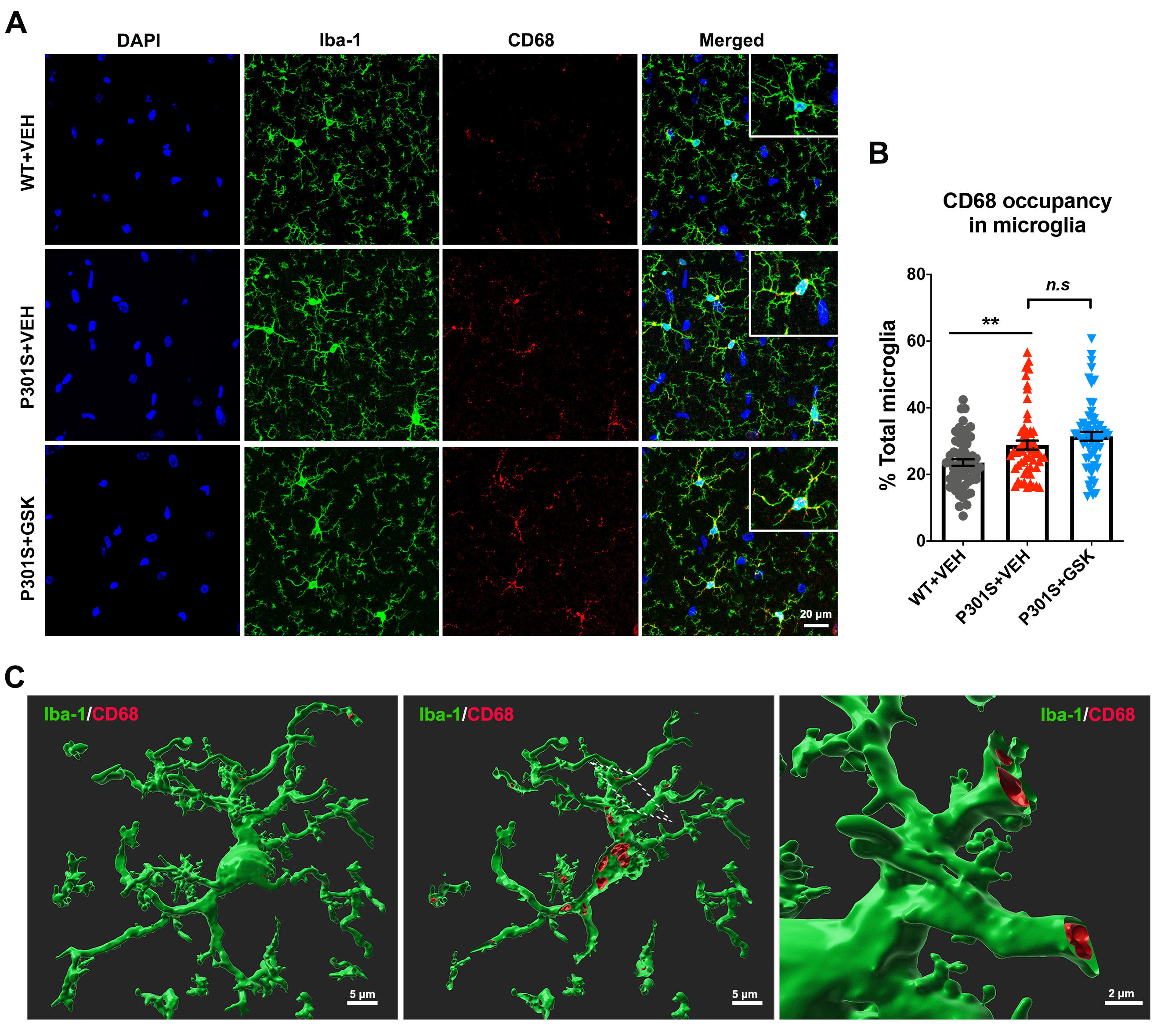

Supplement: Supplementary file 5 — Additional file 5: Supplementary Fig. S5. Effect of GSK1482160 on tau pathology was independent of phagocytosis activity of microglia. A and B. Microglia phagocytic function analysis. Microglia (Iba-1, green) co-stained with a lysosome marker CD68 (red) (A); Quantification of the %CD68+ area in Iba-1+ microglia (B), **p < 0.01, one-way ANOVA with Dunnett’s post hoc, each dot represents an individual cell, n = (61, 59, 61) for (WT + VEH, P301S + VEH, P301S + GSK1482160) from 5 animals per group. C. 3D surface rendering of CD68+ area in microglia from P301S + GSK142160 group by Imaris software. [file 13024_2020_396_MOESM5_ESM.tif]

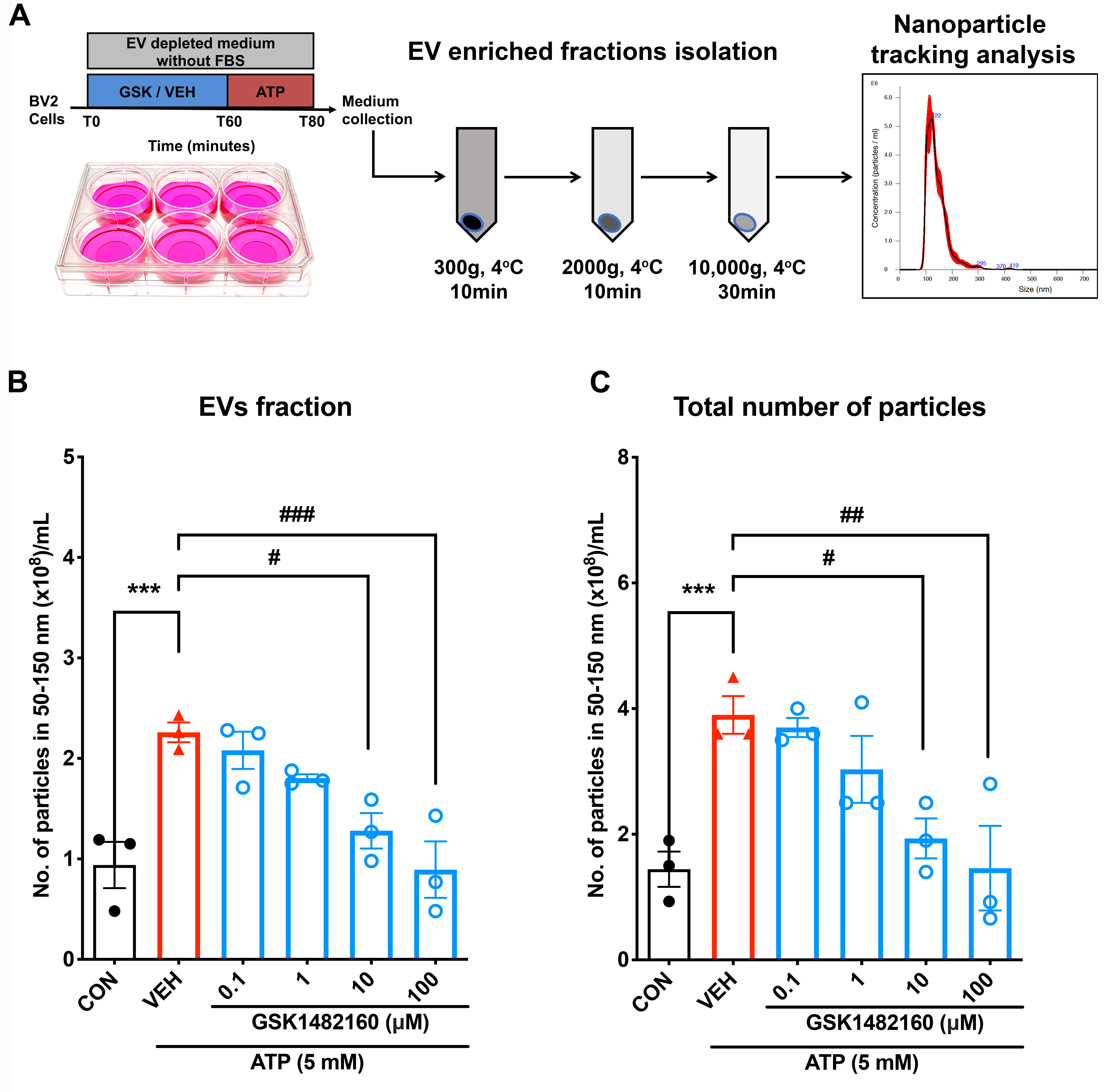

Supplement: Supplementary file 6 — Additional file 6: Supplementary Fig. S6. GSK148620 treatment suppress exosomal secretion from murine microglial-like BV2 cells. A. Scheme of EV isolation protocol from murine microglial cell line BV-2 conditioned media after ATP stimulation. B-C. NTA analysis of isolated EVs. ***p < 0.001, compared with Control group; #p < 0.05, ##p < 0.01 and ###p < 0.01, compared with Vehicle-treated group; n.s denotes no significance as determined by one-way ANOVA (alpha = 0.05) and Dunnett’s post-hoc. Graphs indicate mean ± s.e.m. [file 13024_2020_396_MOESM6_ESM.tif]
