# Supplementary material for: P2RX7 inhibitor suppresses exosome secretion and disease phenotype in P301S tau transgenic mice
Source: Mol Neurodegener. 2020 Aug 18;15:47. doi: 10.1186/s13024-020-00396-2 (PMC7436984; doi:10.1186/s13024-020-00396-2)
Supplement: Supplementary file 8 — Additional file 8. Supplementary Methods Materials and Methods. [file 13024_2020_396_MOESM8_ESM.docx]

**P2RX7 inhibitor suppresses exosome secretion and disease phenotype in P301S tau transgenic mice**

Zhi Ruan^1^, Jean-Christophe Delpech^1^, Srinidhi Venkatesan Kalavai^1^, Alicia A. Van Enoo^1^,

Jianqiao Hu^1^, Seiko Ikezu^1^ and Tsuneya Ikezu^1,2,3,4#^

^1^Department of Pharmacology & Experimental Therapeutics, ^2^Alzheimer’s Disease Center, ^3^Center for Systems Neuroscience, ^4^Neurology, Boston University School of Medicine, 72 East Concord St, L-606B, Boston, MA 02118, USA

**Supplementary Materials and Methods**

**Animals**

The P301S tau transgenic mouse line (P301S, also known as PS19), which expresses human tau (isoform 1N4R, containing four microtubule-binding repeats and lacking exon 3) carrying the FTDP-17-linked P301S mutation [1], was obtained from Jackson Laboratory (B6;C3-Tg(Prnp-MAPT*P301S)PS19Vle/J, #008169). Wild-type (WT) strain B6C3F1 was used to maintain P301S transgene hemizygotes. Mice were kept on a 12h light- 12h dark schedule with access to chow ad libitum. Starting at 3 months of age, mice were randomized to receive GSK1482160 (20 mg/kg) or vehicle by oral gavage two doses per day for 30 days. During the study, both groups gained weight normally. No adverse effect was observed in the animals receiving the drug treatment. For behavior tests, total number of animals used in each group was 17, 21, 20 for WT, P301S+vehicle, P301S+GS1482160, respectively. The numbers of animals for the rest of the experiments were summarized in each figure legend. Although the experiments were not sufficiently powered to detect significant sex differences, nothing overt was observed, and data from males and females in the same genotype/treatment group were combined. All animal procedures followed the guidelines of the National Institutes of Health Guide for the Care and Use of Laboratory Animals and were approved by the Boston University Institutional Animal Care and Use Committee.

**Preparation of GSK1482160 solution**

GSK1482160 (334.7236 g/mol, >97% purity, PharmaBlock Sciences, Inc., CAS 1001389-72-5) was dissolved in dimethyl sulfoxide (DMSO) to the final concentration of 100mM as stock solution. For the animal experiment, GSK1482160 was dissolved in vehicle [1% (w/v) methylcellulose, Millipore Sigma, M0555] in sterilized water at a concentration of 2.0 mg/mL and administered p.o. twice a day at a target dose of 20 mg/kg[2].

**Immunohistochemistry (IHC)**

Brain tissues were removed after transcardial perfusion fixation with 4% paraformaldehyde/PBS, followed by post-fixation and cryoprotection with 30% sucrose/PBS. Fixed frozen brain tissues were coronally sectioned using a cryostat (Thermo Fisher Scientific, Cryostar NX50 OPHV) at 30-µm thickness, and three sections of dorsal hippocampal regions per brain per antibody were used for IHC. The sections were subjected to antigen retrieval with sodium citrate (10mM, pH 8.0) at 80°C for 20 min, then blocked in 10% normal donkey serum, 1% bovine serum albumin (BSA, Millipore Sigma) and 0.1% Tween 20/PBS for 1h. Sections were incubated with mononuclear phagocyte marker ionized calcium-binding adapter molecule 1 (Iba-1, 1:1000, rabbit IgG, #019-19741,Wako), P2ry12(1:100, #848001, rat IgG, Biolegend), NeuN(1:500, #Ab177487, rabbit IgG, Abcam), MAP2(1:50, #PA5-17646, rabbit IgG, Thermofisher), GFAP(1:500, #z0334, Rabbit IgG, Dako), MOG(1:50, #ab32760, rabbit IgG, Abcam), exosome-specific endosomal sorting complex required for transport-1 (ESCRT-1) marker Tsg101 (1: 50, goat IgG, sc-6037 and mouse IgG, sc-101254, Santa Cruz), CD81 (1:100, #LS-C350457, mouse IgG, LSBio), CD68(1:500, #MCA1957, rat IgG, Bio-rad), misfolded tau markers Alz50 (1: 50, mouse IgM) and MC1(1: 400, mouse IgG), both as a kind gift from Dr. Peter Davies, diluted with 1% BSA and 0.02% tween in PBS overnight. The samples were washed with TBST 3 times and incubated in secondary antibodies (donkey anti-rabbit IgG AlexaFluor 488, 1:500; donkey anti-mouse IgM AlexaFluor 594, 1:500; donkey anti-goat IgG AlexaFluor 647, 1:500, Invitrogen) for 1h at room temperature in darkness. For MC1 staining, IHC was performed using the ImmPRESS® HRP Anti-Mouse IgG (Peroxidase) Polymer Detection Kit (Vector Laboratories, MP-7402-50) and 3,3'-diaminobenzidine tetrahydrochloride (Thermo Fisher Scientific, 34001) as substrate.

**Confocal image processing and quantification by Imaris**

All confocal imaging was performed on a LSM710 using Zen 2010 software (Zeiss) or a Leica TCS SP8 lightning microscope at the inverted Leica DMi8 microscope stand using the confocal mode with HC PL APO CS2 40x/1.3 and 63×/1.4  oil immersion objectives. Images of 2048 × 2048 pixels as confocal stacks with a z-interval of 0.20 μm system optimized was used to image cells. For imaging CD68 and CD81, a 552-nm laser line was used and emission was collected at 565–650 nm; for imaging Iba-1, a 488-nm laser line was used and emission was collected at 490-600 nm. All co-localization images were scanned frame by frame in the sequential scanning mode, which showed no cross talk among multiple channels. Gain and off-set were set at values which prevented saturated and empty pixels. After image acquisition, all images were applied with lightning deconvolution. ImageJ was used to perform background subtraction and thresholding. For microglial morphology assays, background subtraction was performed using ImageJ, then Imaris software 9.5, 64-bit version (Bitplane AG, Saint Paul, MN, www.bitplane.com) was used to perform 3D reconstruction and surface rendering [3]. Imaris Filament Tracer were used to quantify the filament total length, branch endpoints, Sholl intersections of individual microglia. Using the resulting tracings, the total process length, number of terminal branch points per process and total intersections of each cell were calculated[4]. Final data analysis was performed using Microsoft Excel and Graph rendering was done in GraphPad Prism.

**Proximity ligation assay (PLA)**

PLA was performed using fixed frozen tissue sections following the protocol from the manufacturer (Duolink, Millipore Sigma, DUO92105). Until the PLA probe incubation step, all manipulations were performed as described in IHC section. Two PLA probes (anti-goat minus and anti-rabbit plus) were tested with the primary antibody pairs (Alz50 mouse monoclonal and anti-Tsg101 goat polyclonal (Santa Cruz, sc-6037) or anti-Hgs rabbit polyclonal (Thermo Fisher Scientific, PA5-27491). Prior to the application, Alz50 was incubated with rabbit or goat anti-mouse IgM secondary antibody (Thermo Fisher Scientific, 31172) at 4^o^C for 1h. All steps for hybridization, enzymatic ligation, and DNA polymerase amplification with fluorescent oligonucleotides were performed at 37^o^C in a humidity chamber, except for the washing steps. Coverslips were then mounted using Duolink *in situ* mounting medium with DAPI. Images were acquired using a Zeiss LSM 880 confocal microscope equipped with Airyscan module, using a 63× objective (Plan-Apochromat, NA 1.4, all from Zeiss).

**Behavioral tests**

All mice were pre-handled for 3 days prior to testing and habituated in the behavior room for 1 h before the test. All tests were performed by experimenters blinded to the treatments.

**Y-maze**

The Y-maze assesses working memory based on the innate preference of a mouse to alternate arms while exploring a new environment. Typically, mice prefer to explore a new arm of the maze rather than returning back to the one that was previously explored. The Y-maze apparatus consisted of three arms 35 cm (length) × 7 cm (height) x 5 cm (width) (San Diego Instruments). Testing was always performed in the same room and at the same time to ensure environmental consistency as previously described[5]. Briefly, each mouse was placed at the end of one arm and allowed to explore freely through the maze during a 10-min session. The sequence and total number of arms entered were video-recorded. An entry into an arm was considered valid if all four paws entered the arm. An alternation was defined as three consecutive entries in three different arms (that is, A, B, C or B, C, A and so on). The percentage alternation score was calculated using the following formula: Total alternation number/total number of entries − 2) × 100. Furthermore, total number of arm entries and total distance moved were used as a measure of general activity in the animals. The maze was cleaned with 20% ethanol after each mouse to minimize odor cues.

**Pre-pulse inhibition (PPI)**

For PPI measurements, an SR-LAB system acoustic startle box with digitized electronic output (San Diego Instruments) containing a piezoelectric accelerometer mounted under a Plexiglas cylinder was used to generate and measure startle response and PPI. Each testing session began with a 5-min acclimation period at background noise intensity of 65 db. This was followed by four pulse alone trials, then a pseudorandom admixture of pulse-alone, pre-pulse + pulse, and no stimulus trials; followed by four more pulse alone trials for a total of 48 trials per test session according to a previously established protocol[6]. For all trials, the SR-LAB machine was programmed to deliver acoustic startle stimuli (or no stimulus) over a background noise level of 65 db with a variable inter-trial interval; the startling stimulus was presented as a fast-rise noise burst lasting 40 ms at an intensity of 120 db. The animal’s whole-body flinch response to each stimulus was recorded as 48 consecutive 250-ms recordings beginning at stimulus onset. For pre-pulse + pulse trials, a pre-pulse of 3, 6, and 12 db over background (68, 71, and 77 db) of 20 ms duration preceded the primary pulse by 100 ms. Baseline startle reactivity was calculated from the average startle magnitude for the initial four pulse alone (120 db) trials from the first PPI session. Pre-pulse inhibition was defined as the percentage of the decline of startle response (pre-pulse inhibition (%) = 100−[(startle amplitude after pre-pulse and pulse)/(startle amplitude after pulse only)×100]).

**Fear conditioning**

The fear conditioning tests were performed as previously described [7,8] with minor modifications. Briefly, the mice were trained and tested in a conditioning chamber (26 × 34 × 29 in cm, Med-Associates Inc.) equipped with black methacrylate walls, transparent front door, a speaker and grid floor. On day one, each mouse was placed into the conditioning chamber for learning. Baseline freezing was quantified during the initial 4-min period. Beginning at 4 min and at 120s intervals thereafter, the mouse was exposed to 3 times to a 0.75-mA continuous foot shock (unconditioned stimulus; US). Broadband white noise was used instead of a frequency-specific tone in an effort to avoid possible auditory deficits that might occur with age. The mouse was removed from the chamber 1 min after the last foot shock, and placed back in its home cage. The contextual fear-conditioning memory was tested 24 h after the training phase, when the animal was placed back inside the conditioning chamber for 5 min without any shock. The freezing responses to the environmental context were quantified over a 5-min period to evaluate contextual fear conditioning.

**Primary tissue culture of murine microglia, astrocyte and cortical neurons**

Primary cultures of mouse microglia were prepared from CD-1 mouse P0 pups as described [9]. Microglia were isolated using a magnetic CD11b bead (Miltenyi Biotec, 130-049-601) separation technique as previously described [10]. Microglia were maintained with DMEM medium (Invitrogen, 11965118) with 10% FBS (Invitrogen, 10082147) in a 5% CO_2_ humidified incubator. Murine monocyte colony stimulating factor (10 ng/ml, cat. no. 4238, BioVision, Milpitas, CA) was added for microglial culture after plating. Purified recombinant 2N4R human tau 1–441 (hTau, 500 μg/ml, cat. no. T-1001-1, rPeptide, Bogart, GA) was preincubated with 30 μM heparin (cat. no. 07980, Stemcell Technologies, Vancouver, Canada) for 72 h before added into microglia on days *in vitro* (DIV) 3 for 24 hours. Murine primary cortical neurons and astrocyte were prepared from E16 P301S mouse embryonic brains as described [11] and brain tissue was dissociated with 0.25% Trypsin-EDTA (Thermo Fisher, 25200072) into single cell. Neurons were maintained in neurobasal medium (Thermo Fisher, 21103049) with 2% B27 supplement (Thermo Fisher, 17504044) and 2 mM L-glutamine (Thermo Fisher, 25030149) at 37^o^C for 14 days. Glial cell growth was inhibited by adding of 5-fluoro-2'-deoxyuridine (30 μM, Sigma-Aldrich, F0503) into the culture from DIV4 [12]. Astrocyte were maintained in DMEM (Invitrogen, 11965118) containing 10% FBS (Invitrogen, 10082147), 1 mM of sodium pyruvate (Invitrogen, 11360070) and GlutaMax-I (Invitrogen, 35050061) at 37^o^C for 14 days [13].

**EVs enriched fraction isolation**

The cells were washed twice with double filtered-PBS to remove serum factors and medium was replaced with GSK1482160 or vehicle in one mL conditioned medium (CM), which was freshly-prepared with 0.45-µm PE membrane double-filtered DMEM medium. Cells were incubated at 37^o^C for another 1h before exposed to 3 hours of 1 μg/mL of LPS followed with 5 mM ATP stimulation for 15 min. CM was collected and enriched for exosomes by sequential high-speed centrifugation as described[11]. Briefly, the CM was centrifuged at 300 × *g* for 10 min, and the supernatant was centrifuged at 2,000 × *g* for 10 min at 4°C to remove cell debris. The supernatant was centrifuged at 10,000 × *g* for 30 min at 4°C to remove microvesicles. The supernatant was ultracentrifuged at 100,000 × *g* for 70 min at 4°C to enrich EVs fraction in pellets. With our purification method, enriched EVs under ATP stimulation were not positive for apoptotic markers[14] nor contaminated by intracellular organelles derived from damaged cells [15]. The EVs-enriched fraction was collected and stored at -80^o^C until further use.

**Nanoparticle Track Analysis (NTA)**

NTA was performed with a NanoSight NS300 (Malvern Panalytical), equipped with a sample chamber and an sCMOS camera. EV samples were diluted in double-filtered PBS to adjust the particle numbers to the optimal range (10–100 particles/frame). Particle live-imaging settings were set according to the manufacturer’s software manual (NanoSight, NS300 User Manual, MAN0541-01-EN-00, 2017).

**Tau and CD9 ELISA**: Mouse hippocampal and cortical tissues were dissected under the dissection microscope (Nikon Instruments) after transcardial perfusion with ice-cold PBS, snap frozen in dry ice and stored at -80^o^C. Tissue samples preparation and all the ELISA applications followed the manufacture’s protocol: Tau (Total) Human ELISA Kit (KHBO042), Tau [pT181] Human ELISA Kit (KHBO0631), Tau (Phospho) [pS396] Human ELISA Kit (KHB7031), Tau (Phospho) [pS199] Human ELISA Kit (KHB7041) and Tau (Phospho) [pT231] Human ELISA Kit (KHB8051) (all from Thermo Fisher Scientific). For CD9 ELISA, custom ELISA kits were developed according to the manufacturer’s instruction using anti-CD9 mouse monoclonal antibody (EMD Millipore, CBL162MI) and biotinylated anti-CD9 antibody using the Antibody Biotinylation Kit (Pierce/Thermo Fisher Scientific, 90407)[16]. The ELISA method consisted of capture and detection antibodies, streptavidin Poly-HRP (Pierce/Thermo Fisher, 21140), TMB solution (Thermo Fisher Scientific, N301) and Stop solution (Thermo Fisher Scientific, N600) and OD 450nm was read by a microplate reader (BioTek Instruments). Each EV sample (100 μL/well) was tested using CD9 ELISA in duplicates.

**Real-time PCR analysis of *Il6*, *Il10*, *Tgfb1*, *Il1a*, *Il1b*, *Il18*, *Ifng* and *Tnfa* mRNA**

Total RNA was isolated from frozen hippocampus tissue with Trizol (Invitrogen) and was reverse transcribed using SuperScript™ IV VILO™ Master Mix kit (Invitrogen, #11766050), and resulting cDNAs were diluted 5 fold before used. To evaluate the level of gene expression, real-time PCR with SYBR Green dye was applied. For mouse *Il6*, the forward and reverse sequences were CTGCAAGAGACTTCCATCCAG and AGTGGTATAGACAGGTCTGTTGG, respectively. For mouse *Il10*, the forward and reverse sequences were CTTACTGACTGGCATGAGGATCA and GCAGCTCTAGGAGCATGTGG, respectively. For mouse *Tgfb1*, the forward and reverse sequences were CCACCTGCAAGACCATCGAC and CTGGCGAGCCTTAGTTTGGAC, respectively. For mouse *Il1a*, the forward and reverse sequences were TCTCAGATTCACAACTGTTCGTG and AGAAAATGAGGTCGGTCTCACTA, respectively. For mouse *Il1b*, the forward and reverse sequences were GAAATGCCACCTTTTGACAGTG and TGGATGCTCTCATCAGGACAG, respectively. For mouse *Il18*, the forward and reverse sequences were GTGAACCCCAGACCAGACTG and CCTGGAACACGTTTCTGAAAGA, respectively. For mouse *Ifng*, the forward and reverse sequences were GCCACGGCACAGTCATTGA and TGCTGATGGCCTGATTGTCTT, respectively. For mouse *Tnfa*, the forward and reverse sequences were CAGGCGGTGCCTATGTCTC and CGATCACCCCGAAGTTCAGTAG, respectively. The PCR reaction was performed in 5 μL final volume using 386-well plates. The reaction mixture contained 2.5 μL Applied Biosystems™ SYBR™ Green PCR Master Mix (Fisher Scientific, #4309155), 100 nM of each primer (forward and reverse) and 2 μL cDNA. All samples were run in duplicate. After sealing plates, mixing wells, and spinning plates at 1000 x g for 4 minutes, 40 cycles of amplification was performed with the standard program according to manufacturer’s specifications in an Applied Biosystems 7900HT qRT-PCR machine. Relative amounts of mRNA were calculated as the comparative C*t* after normalized to the GAPDH control C*t* value.

**Statistical Analyses**

All data are presented as means ± standard error of the mean (s.e.m). Comparisons between two groups were done by two-tailed paired or unpaired Student’s *t*-tests. Multiple comparisons were performed by either one- or two-way ANOVA, followed by Bonferroni’s or Dunnett’s *post hoc*. Statistical analyses were performed using Prism 8.0 (GraphPad Software). A statistically significant difference was assumed at p <0.05.

**References**

1 Yoshiyama, Y. *et al.* Synapse loss and microglial activation precede tangles in a P301S tauopathy mouse model. Neuron. 2007;53: 337-351.

2 Abdi, M. H. *et al.* Discovery and structure-activity relationships of a series of pyroglutamic acid amide antagonists of the P2X(7) receptor. Bioorg Med Chem Lett. 2010;20: 5080-5084.

3 Schafer, D. P. *et al.* Microglia sculpt postnatal neural circuits in an activity and complement-dependent manner. Neuron. 2012;74: 691-705.

4 Nayak, D. *et al.* Type I interferon programs innate myeloid dynamics and gene expression in the virally infected nervous system. PLoS Pathog. 2013;9: e1003395.

5 Chu, J. *et al.* Pharmacologic blockade of 12/15-lipoxygenase ameliorates memory deficits, Abeta and tau neuropathology in the triple-transgenic mice. Mol Psychiatry. 2015;20: 1329-1338.

6 Koppel, J. *et al.* Pathogenic tau species drive a psychosis-like phenotype in a mouse model of Alzheimer's disease. Behav Brain Res. 2014;275: 27-33.

7 Sato, C. *et al.* Loss of RBPj in postnatal excitatory neurons does not cause neurodegeneration or memory impairments in aged mice. PLoS One. 2012;7: e48180.

8 Wozniak, D. F., Xiao, M., Xu, L., Yamada, K. A. & Ornitz, D. M. Impaired spatial learning and defective theta burst induced LTP in mice lacking fibroblast growth factor 14. Neurobiol Dis. 2007;26: 14-26.

9 Sarkar, S. *et al.* Mitochondrial impairment in microglia amplifies NLRP3 inflammasome proinflammatory signaling in cell culture and animal models of Parkinson's disease. NPJ Parkinsons Dis. 2017;3: 30.

10 Sarkar, S. *et al.* Rapid and Refined CD11b Magnetic Isolation of Primary Microglia with Enhanced Purity and Versatility. J Vis Exp. 2017;122:55364.

11 Asai, H. *et al.* Depletion of microglia and inhibition of exosome synthesis halt tau propagation. Nat Neurosci. 2015;18: 1584-1593.

12 Xiong, Y. L. *et al.* GTPase Activity Plays a Key Role in the Pathobiology of LRRK2. Plos Genet. 2010;6:e1000902.

13 Yun, S. P. *et al.* Block of A1 astrocyte conversion by microglia is neuroprotective in models of Parkinson's disease. Nature Medicine. 2018;24: 931-938.

14 Verderio, C. *et al.* Myeloid microvesicles are a marker and therapeutic target for neuroinflammation. Ann Neurol. 2012;72: 610-624.

15 Gabrielli, M. *et al.* Active endocannabinoids are secreted on extracellular membrane vesicles. EMBO Rep. 2015;16: 213-220.

16 Khodashenas, S., Khalili, S. & Moghadam, M. F. A cell ELISA based method for exosome detection in diagnostic and therapeutic applications. Biotechnol Lett. 2019;41: 523-531.
